# Bulk Spin–Orbit Torque-Driven Spin Hall Nano-Oscillators Using PtBi Alloys with Engineered Crystallinity

**DOI:** 10.1021/acsami.6c06092

**Published:** 2026-07-13

**Authors:** Utkarsh Shashank, Akash Kumar, Tahereh Sadat Parvini, Hauke Heyen, Lunjie Zeng, Andrew B. Yankovich, Jong-Guk Choi, Mona Rajabali, Eva Olsson, Markus Münzenberg, Johan Åkerman

**Affiliations:** † Applied Spintronics Group, Department of Physics, 3570 University of Gothenburg, Gothenburg 412 96, Sweden; ‡ Research Institute of Electrical Communication, Tohoku University, 2-1-1 Katahira, Aoba-Ku, Sendai 980-8577, Japan; § Center for Science and Innovation in Spintronics, Tohoku University, 2-1-1 Katahira, Aoba-Ku, Sendai 980-8577, Japan; ∥ Institut für Physik, 130432 Universität Greifswald, Greifswald 17489, Germany; ⊥ Walther-Meißner-Institut, Bayerische Akademie der Wissenschaften, Garching 85748, Germany; # Munich Center for Quantum Science and Technology (MCQST), Schellingstr. 4, Munich 80799, Germany; ∇ Department of Physics and Astronomy, 11248 Chalmers University of Technology, Gothenburg 412 96, Sweden; ○ NanOsc AB, Kista 164 40, Sweden

**Keywords:** spin−orbit torque, spin Hall effect, spin Hall nano-oscillator, auto-oscillation, extrinsic side-jump scattering

## Abstract

Spin–orbit-torque-driven auto-oscillations in spin Hall nano-oscillators (SHNOs) provide a promising route toward energy-efficient, nanoscale microwave devices for neuromorphic computing and high-frequency technologies. Achieving robust oscillations requires lowering the threshold current (*I*_th_), governed by the spin Hall efficiency (θ_SH_). Conventional approaches to enhance θ_SH_ often involve trade-offs, such as increased resistivity and interfacial effects. Here, we demonstrate a pronounced enhancement of the bulk spin Hall effect in PtBi alloys via crystallographic engineering, achieving a 3-fold increase in θ_SH_ from 0.07 in Pt_100.0_Bi_0.0_ to 0.24 in Pt_94.0_Bi_6.0_ and 0.19 in Pt_91.3_Bi_8.7_, extracted from DC-bias spin-torque ferromagnetic resonance. The enhancement arises from bulk-dominated extrinsic side-jump scattering. Correspondingly, *I*_th_ is reduced by 42% and 32% in 100 nm SHNOs based on Co_40_Fe_40_B_20_(3 nm)/Pt_94.0_Bi_6.0_(4 nm) and Co_40_Fe_40_B_20_(3 nm)/Pt_91.3_Bi_8.7_(4 nm), respectively. The devices exhibit narrower linewidths (∼25 MHz), enhanced quality factors (350 ≤ *Q* ≤ 550, ∼4× higher than Pt (Pt_100.0_Bi_0.0_)), and a 61.6% reduction in threshold power. These findings establish PtBi alloys as efficient spin Hall materials, enabling reduced power consumption for SHNO-based neuromorphic and memory technologies.

## Graphical abstract

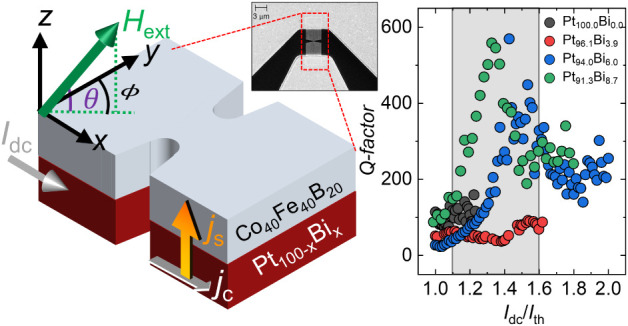

## Introduction

The efficient control of magnetization via spin–orbit torque (SOT),^1^ primarily generated by the Spin Hall effect (SHE),^2,3^ lies at the core of modern spintronics.^4^ It plays a key role in magnetization switching,^5–8^ in the generation and control of propagating spin waves,^9,10^ in spin Hall nano-oscillators (SHNOs)^11,12^ and in the movement of domain walls and skyrmions.^13–15^ In a heavy metal (HM) with large spin–orbit coupling, the SHE generates a sizable transverse spin current density, **j**_s_, from a longitudinal charge current density, **j**_c_. The generated **j**_s_ can exert different torques on the magnetization of an adjacent ferromagnet (FM) layer, where the damping-like torque, **τ**_DL_, is the component collinear with the intrinsic damping torque, **τ**_α_, of the FM layer.^16^ If **τ**_DL_ balances **τ**_α_, the threshold condition for FM auto-oscillations (AO), with a corresponding threshold current (*I*_th_), is reached in the SHNO.^17,18^ Over the past decade, SOT-driven nanoconstriction-based SHNOs have garnered significant attention thanks to their straightforward nanofabrication, direct optical and gate access to the auto-oscillating FM region, and rich magneto-dynamical behavior.^19^ SHNOs have shown tremendous promise for mutual synchronization in chains^20,21^ and arrays,^22–24^ paving the way for applications in neuromorphic computing,^25–27^ Ising machines,^28^ magnonic conduits,^10^ and high-frequency GHz technologies.^29^

To reduce *I*_th_, a higher conversion of **j**_c_ to **j**_s_, defined as the spin Hall efficiency, θ_SH_, is required. θ_SH_ can be expressed as $j_{s}=\frac{\hbar }{2e}\theta_{\mathrm{SH}}\left(j_{c}\times \mathrm{\sigma ̂}\right)$ , where ***σ̂*** is the polarization of the spin current, *e* is the elementary charge, and *ℏ* is the reduced Planck constant. The most commonly used HM, Pt, has a moderate θ_SH_ ≈ 0.05–0.09, arising from its intrinsic SHE.^16,30–32^ Ion implantation^33–36^ or sputtering^37–39^ of nonmetallic, lighter impurities, such as S, O, N, and P, can also be used to tune the θ_SH_ of Pt, Ta, and W. However, such tuning requires careful materials engineering and often comes with a trade-off in the form of increased longitudinal resistivity (ρ_*xx*_), as observed in TaN, which can exhibit values as high as 3000 μΩ·cm.^39^ In contrast, metallic impurities introduced by cosputtering with the HM neither require complex engineering nor introduce drastic changes in ρ_*xx*_. A better trade-off between θ_SH_ and ρ_*xx*_ is crucial for low-power consumption in SOT magnetic random-access memory (SOT-MRAM).^36,40^ In this context, Pt-, Ta-, and W-based alloys have been explored (θ_SH_ values in parentheses), such as Pt_28_Cu_72_ (0.07),^41^ Pt_75_Pd_25_ (0.26),^42^ Pt_90_Pd_10_ (0.17),^43^ Pt_92_Bi_8_ (0.10),^44^ Pt_88_Mo_12_ (0.35),^45^ Pt_70_Gd_30_ (0.27),^46^ Pt_75_Au_25_ (0.35),^47^ Pt_53_Au_47_ (0.32);^48^ W_100–*x*_Ta_*x*_ (−0.35 to −0.62),^49^ and Cu_100–*x*_Ta_*x*_ (−0.04 to −0.09).^39^ Within this framework, Hayashi et al. showed that Pt_100–*x*_Bi_*x*_/Co bilayers with substantial Bi concentrations (*x* = 25–50) exhibit markedly improved θ_SH_ and reduced switching current densities relative to Pt, identifying PtBi as a strong candidate for energy-efficient magnetization switching.^50^ Complementing this, Münzenberg et al. demonstrated that Pt_92_Bi_8_-based heterostructures can act as high-performance THz spintronic emitters, delivering broader bandwidths and higher central frequencies than conventional bilayer systems.^51^ In addition, at higher Bi concentrations, such as in stoichiometric PtBi_2_, this material has attracted attention as a topological system, exhibiting giant three-dimensional Rashba-like spin splitting (α_R_ ≈ 4.36 eV·Å)^52^ and surface-bound superconducting gaps reaching up to 20 meV.^53^ However, when the Bi content exceeds *x* ≥ 60%, Pt_100–*x*_Bi_*x*_ alloys transition toward the PtBi_2_ phase,^50^ leading to degraded spin Hall conductivity $\left(\sigma_{\mathrm{SH}}^{xy}\right)$ due to increased ρ_*xx*_, which causes significant current shunting into the FM layer, resulting in Joule heating and deterioration of spectral quality in SHNOs, as illustrated in our previous work.^22^ Therefore, optimization at moderate Bi levels is paramount for attaining practical device applications, especially SHNOs. Furthermore, despite these advances, a unified understanding of the reciprocal connection between θ_SH_ and *I*_th_ in Pt_100–*x*_Bi_*x*_ alloys via both ST-FMR and direct AO measurements remains unexplored.

Here, we report on a dramatic increase in θ_SH_ by more than three times when alloying Pt with Bi, and a corresponding decrease of *I*_th_ by 42% in SHNOs based on Pt_94.0_Bi_6.0_ and Co_40_Fe_40_B_20_. We deposited Pt_100–*x*_Bi_*x*_ alloy thin films using electron-beam coevaporation and systematically varied the Bi concentrations across *x* = 0.0, 3.9, 6.0, and 8.7 (hereafter denoted as Pt_100.0_Bi_0.0_, Pt_96.1_Bi_3.9_, Pt_94.0_Bi_6.0_, and Pt_91.3_Bi_8.7_, respectively). Structural characterization by grazing-incidence X-ray diffraction (GIXRD) and transmission electron microscopy (TEM) reveals a progressive loss of crystallinity with increasing Bi content, suggesting enhanced scattering contributions via crystallographic engineering. Scanning transmission electron microscopy (STEM) and energy-dispersive X-ray spectroscopy (EDXS) investigation indicates a uniform distribution of Bi in Pt. Linewidth analysis of DC-biased spin-torque ferromagnetic resonance (ST-FMR) measurements vs current yields a sharp increase in θ_SH_ from 0.07 ± 0.01 in Pt_100.0_Bi_0.0_ to 0.24 ± 0.02 and 0.19 ± 0.01 in the Pt_94.0_Bi_6.0_ and Pt_91.3_Bi_8.7_ alloys, respectively. To rule out artifacts and confirm the bulk origin of SOT, we demonstrate a clear sin 2ϕ cos ϕ in-plane angular dependence of the SOT for all PtBi alloys. Thanks to the increase in θ_SH_, we observe a 42% and 32% reduction in *I*_th_ for Pt_94.0_Bi_6.0_ and Pt_91.3_Bi_8.7_-based 100 nm SHNOs. These PtBi-based SHNOs further exhibit narrower linewidth (Δ*f*) (≈ 25 MHz), enhanced quality factors of ≈350 ≤ *Q* ≤ 550, about ∼4× higher than Pt_100.0_Bi_0.0_, and lower normalized input power (61.6% reduction). Our findings of a higher θ_SH_ driven by bulk-extrinsic SHE, accompanied by a reduced *I*_th_, establish Bi alloying in Pt as a robust strategy for engineering energy-efficient SHNOs, opening new avenues beyond conventional 5*d* transition metals.

## Results and Discussion

### Structural Characterization via GIXRD and TEM

Figure 1a shows out-of-plane grazing-incidence X-ray diffraction (GIXRD) patterns of Pt_100–*x*_Bi_*x*_ thin films (see Experimental Section). Reflections from the (111), (200), (220), and (311) planes confirm a crystalline face-centered cubic (*fcc*) phase in Pt_100.0_Bi_0.0_ and Pt_96.1_Bi_3.9_.^36,54^ Increasing the Bi content reduces the overall reflection intensities, particularly that of the (220) peak, indicating a reduction in crystallinity.^36,55^ Peak broadening becomes more pronounced in Pt_94.0_Bi_6.0_ and Pt_91.3_Bi_8.7_. The intensity ratio *I*_(111)_/*I*_(220)_ rises from 0.385 (0% Bi) to 1.645 (6% Bi), then falls to 1.070 at 8.7% Bi. Similarly, the out-of-plane lattice parameter increases from 3.9525 to 3.9653 Å (0–6% Bi), followed by a slight decrease to 3.9595 Å at 8.7% Bi. This nonmonotonic trend suggests a shift in preferred crystallographic orientation, likely due to strain-induced texturing at higher Bi concentrations, consistent with previous reports.^50^ The extracted out-of-plane lattice parameters and texture-related peak intensity ratios are summarized in Table 1.

**Figure 1:**
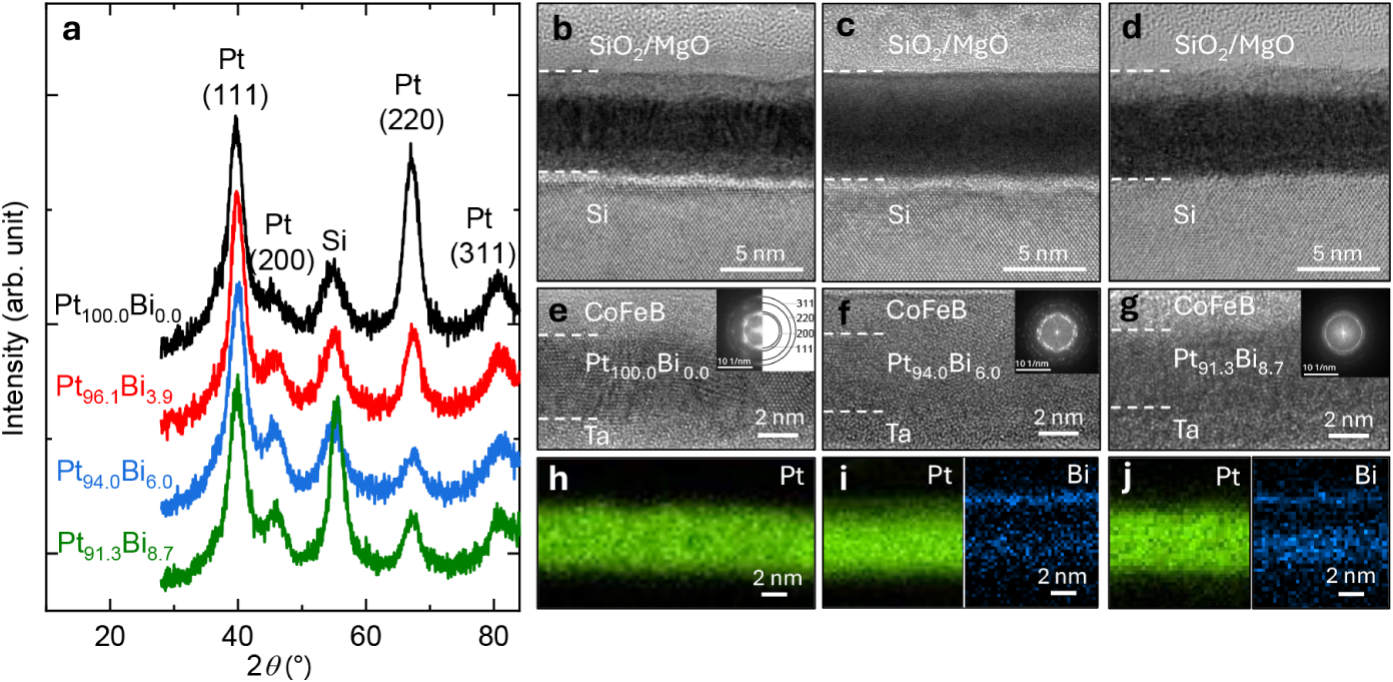
(a) GIXRD patterns of the Pt_100.0_Bi_0.0_ stack (black) and PtBi alloy stacks with compositions Pt_96.1_Bi_3.9_ (red), Pt_94.0_Bi_6.0_ (blue), and Pt_91.3_Bi_8.7_ (green). (b–d) Cross-sectional TEM images of (b) the Pt_100.0_Bi_0.0_ stack (HR-Si/Ta­(2.4 nm)/Pt­(4.0 nm)/Co_40_Fe_40_B_20_(3.0 nm)/MgO­(2.0 nm)/SiO_2_(3.0 nm)), (c) the Pt_94.0_Bi_6.0_ stack (HR-Si/Ta­(2.4 nm)/Pt_94.0_Bi_6.0_(4.0 nm)/Co_40_Fe_40_B_20_(3.0 nm)/MgO­(2.0 nm)/SiO_2_(3.0 nm)), and (d) the Pt_91.3_Bi_8.7_ stack (HR-Si/Ta­(2.4 nm)/Pt_91.3_Bi_8.7_(4.0 nm)/Co_40_Fe_40_B_20_(3.0 nm)/MgO­(2.0 nm)/SiO_2_(3.0 nm)), all showing uniform, well-defined layers with comparable total thicknesses. (e–g) High-resolution TEM images of the same stacks with diffractograms obtained from HR-TEM images in respective insets. (e) The Pt_100.0_Bi_0.0_ stack shows clear lattice fringes and sharp Bragg spots (inset diffractogram), indicative of a crystalline Pt structure. The distinct and indexed (220) and (111) spots confirm the crystalline face-centered cubic (fcc) nature of the Pt_100.0_Bi_0.0_ layer. (f, g) The Pt_94.0_Bi_6.0_ and Pt_91.3_Bi_8.7_ stacks display more disordered lattice fringes and reduced intensity of higher order (220 and 311) diffraction rings in the diffractograms (insets), suggesting lower crystallinity. (h–j) STEM-EDXS elemental maps of Pt and Bi for the Pt_100.0_Bi_0.0_, Pt_94.0_Bi_6.0_, and Pt_91.3_Bi_8.7_ stacks, respectively. Bi is uniformly distributed within the Pt_94.0_Bi_6.0_ and Pt_91.3_Bi_8.7_ layers, with enrichment at the PtBi/Co_40_Fe_40_B_20_ interface. Additional HAADF-STEM images and STEM-EDXS elemental maps and line profiles are provided in Figures S1 and S2.

**Table 1:** Extracted Out-of-Plane Lattice Parameters and Relative Peak Intensity Ratios Obtained from GIXRD Measurements of Pt_100–*x*_Bi*_x_* Films^a^

Composition	I(111)/I(220)	Lattice parameter, *a* (Å)
Pt_100.0_Bi_0.0_	0.385	3.9525
Pt_96.1_Bi_3.9_	0.852	3.9558
Pt_94.0_Bi_6.0_	1.645	3.9653
Pt_91.3_Bi_8.7_	1.070	3.9595

a The variation in the I(111)/I(220) ratio indicates changes in crystallographic texture with increasing Bi concentration.

Figure 1b–d displays cross-sectional TEM images of the Pt_100.0_Bi_0.0_ stack, the Pt_94.0_Bi_6.0_ stack, and the Pt_91.3_Bi_8.7_ stack, respectively. All stacks exhibit well-defined, uniform layered structures with comparable total thicknesses. Figure 1e–g presents HRTEM images of the samples. In the Pt_100.0_Bi_0.0_ (Figure 1e), clear lattice fringes are visible, and the diffractogram (inset) shows distinct Bragg spots and rings, indicating a crystalline structure of the Pt layer.^33,36^ In contrast, the Pt_94.0_Bi_6.0_ sample (Figure 1f) exhibits more disordered lattice fringes and diminished Bragg spots and higher-order (220 and 311) rings (inset diffractogram), suggesting reduced crystallinity, consistent with the GIXRD results. Furthermore, for the Pt_91.3_Bi_8.7_ sample (Figure 1g), the lattice fringes become faintest, and the Bragg spots are further diminished, indicating an even greater reduction in crystallinity.^36^ Figure 1h–j provides STEM-EDXS elemental maps for the same samples. In both the Pt_94.0_Bi_6.0_ and Pt_91.3_Bi_8.7_ stacks, Bi is uniformly distributed throughout the target Pt layer, with no sign of clustering. An enrichment of Bi is also observed at the Pt_94.0_Bi_6.0_/Co_40_Fe_40_B_20_ and Pt_91.3_Bi_8.7_/Co_40_Fe_40_B_20_ interfaces. The impact of both bulk and interfacial Bi distributions on the SHE in Pt is addressed in subsequent sections.

### Spin-Torque Ferromagnetic Resonance Measurements

To evaluate how Bi doping modulates the SHE in Pt, we performed spin-torque ferromagnetic resonance (ST-FMR) measurements^16^ (see Experimental Section for details). Figure 2a illustrates the schematic of the ST-FMR measurement and Figure 2b shows the experimental setup. An in-plane microwave current *I*_rf_ with a power of 4 dBm was applied along the *x*-axis of the HM/FM bilayer via a ground-source-ground (GSG) coplanar waveguide (CPW). The external in-plane magnetic field *H*_ext_ was swept ± 500 mT at an angle ϕ = 70° between *I*_rf_ and *H*_ext_. The charge current density **j**_*c*_ (along *x*) driven by *I*_rf_ generates a spin current density **j**_*s*_ along *z* with spin polarization ***σ̂*** along −*y*. This **j**_s_ exerts an in-plane **τ**_DL_ on the magnetization **m** of the FM layer. Simultaneously, the Oersted field, *h*_rf_, generated by *I*_rf_ acts as an out-of-plane Oersted field torque, **τ**_OF_. Interfacial effects, rather than bulk mechanisms, can also induce out-of-plane field-like torques (**τ**_FL_) arising from the Rashba effect, particularly in thinner FM layers (≲1 nm) interfaced with HM,^56^ which may overlap with **τ**_OF_. When **τ**_FL_ is small, as in bulk SHE heterostructures, the combined torques (**τ**_DL_ and **τ**_OF_) govern the magnetization precession. The precession of **m** around the effective field *H*_eff_ induces a time-dependent resistance variation via the anisotropic magnetoresistance (AMR), Δ*R* ∝ cos^2^ ϕ (see Figure S3). Mixing with *I*_rf_, the varying Δ*R* generates a rectified ST-FMR voltage, *V*_dc_. The *I*_rf_ was amplitude modulated at 98.76 Hz, serving as a reference signal for lock-in detection.

**Figure 2:**
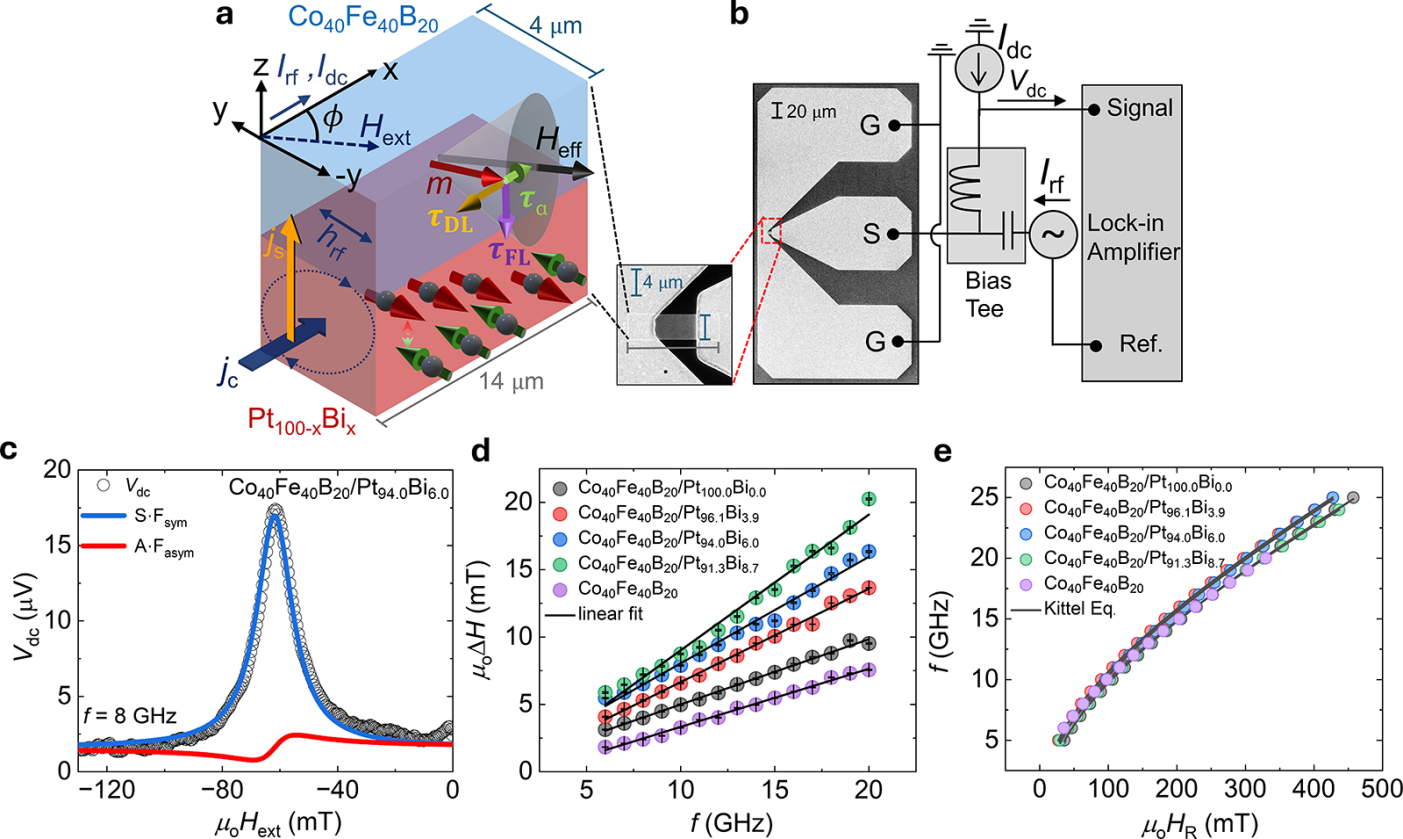
(a) Schematic of the SHE and the microscopic mechanism of the ST-FMR excitation, depicting the torques acting on the FM magnetization. (b) The ST-FMR measurement technique and detection principle along with the microbar device connected to a GSG-CPW. A zoom-in SEM image of the red square shows the microbar. (c) ST-FMR voltage (*V*_dc_) at *f* = 8 GHz of Pt_94.0_Bi_6.0_, with separate symmetric and antisymmetric components fit to eq 1. (d) Δ*H* vs *f* and (e) *f* vs *H*_R_ for *f* = 6–20 GHz, with solid black lines being linear fits to eq 2 for all samples evaluated in this study, including Co_40_Fe_40_B_20_. Solid lines in (e) represent fits to the Kittel eq 3.

Figure 2c shows the ST-FMR voltage spectrum measured at frequency *f* = 8 GHz and *I*_dc_ = 0 mA for Pt_94.0_Bi_6.0_. The measured *V*_dc_ is defined as^16^

1 $$V_{\mathrm{dc}}=SF_{\mathrm{sym}}\left(H_{\mathrm{ext}}\right)+AF_{\mathrm{asym}}\left(H_{\mathrm{ext}}\right)$$

where $F_{\mathrm{sym}}\left(H_{\mathrm{ext}}\right)=\frac{{\left(\Delta H\right)}^{2}}{{\left(\mu_{0}H_{\mathrm{ext}}-\mu_{0}H_{R}\right)}^{2}+{\left(\Delta H\right)}^{2}}$ is the symmetric component, and $F_{\mathrm{asym}}\left(H_{\mathrm{ext}}\right)=\frac{\Delta H\left(\mu_{0}H_{\mathrm{ext}}-\mu_{0}H_{R}\right)}{{\left(\mu_{0}H_{\mathrm{ext}}-\mu_{0}H_{R}\right)}^{2}+{\left(\Delta H\right)}^{2}}$ is the antisymmetric component. The parameters *S* and *A* are the weight factors corresponding to the symmetric and antisymmetric components, respectively. Δ*H* and *H*_R_ are the linewidth (half-width-at-half-maximum) and resonance field of the ST-FMR spectra, respectively. Strikingly, a very high symmetric component *SF*_sym_(*H*_ext_), confirming the high **τ**_DL_, is observed for Pt_94.0_Bi_6.0_. Furthermore, in-plane angular ST-FMR measurements reveal that both the *S* and *A* weight factors exhibit a clear sin 2ϕ cos ϕ dependence across all Pt_100–*x*_Bi_*x*_ compositions (see Figure S4 for details). This angular dependence is characteristic of a conventional bulk-SOT origin, free from torque symmetry breaking, and rules out significant contributions from experimental artifacts.^36,57–60^

Next, we extract the Gilbert damping parameter α_total_ by plotting Δ*H* as a function of *f* (see Figure 2d), using the relation

2 $$\Delta H=\Delta H_{0}+\frac{2\pi \alpha_{\mathrm{total}}}{\gamma \mu_{0}}f$$

where Δ*H*_0_ denotes the frequency-independent inhomogeneous linewidth broadening,^61^ and γ is the gyromagnetic ratio. A systematic increase in α_total_ is observed from 0.0120 ± 0.0004 (Co_40_Fe_40_B_20_) to 0.0140 ± 0.0005 (Co_40_Fe_40_B_20_/Pt_100.0_Bi_0.0_) to 0.0200 ± 0.0007, 0.0230 ± 0.0010, and 0.0290 ± 0.0020 for Co_40_Fe_40_B_20_/Pt_96.1_Bi_3.9_, Co_40_Fe_40_B_20_/Pt_94.0_Bi_6.0_, and Co_40_Fe_40_B_20_/Pt_91.3_Bi_8.7_, respectively. This trend reflects enhanced spin current generation, potentially arising from spin pumping^44^ and/or an increase in SHE.^34,35^ The extracted Δ*H*_0_ remains low and positive (0.1054–0.5675 mT, see Table 2), indicating excellent film quality.^33,34^ Figure 2e depicts the frequency dependence of the resonance field, *H*_R_, using the Kittel formula:

3 $$f=\frac{\gamma \mu_{0}}{2\pi }\sqrt{\left(H_{R}+H_{K}\right)\left(H_{R}+H_{K}+M_{\mathrm{eff}}\right)}$$

where *H*_K_ is the in-plane magnetic anisotropy field and *M*_eff_ is the effective demagnetization.^62^ The extracted μ_0_ *M*_eff_ values, 1038.91 mT (Co_40_Fe_40_B_20_ without Pt or PtBi layer), 1141.38 mT (Pt_100.0_Bi_0.0_), 1268.79 mT (Pt_96.1_Bi_3.9_), 1268.43 mT (Pt_94.0_Bi_6.0_), and 1088.84 mT (Pt_91.3_Bi_8.7_), along with *H*_K_ values of 2.75–12.17 mT, confirm no significant variation in magnetic properties upon Bi incorporation. The extracted magnetic and linewidth fitting parameters are summarized in Table 2.

**Table 2:** Extracted Magnetic and Linewidth Fitting Parameters Obtained from ST-FMR Measurements^a^

Stack	α_total_	Δ*H*_0_ (mT)	μ_0_ *M*_eff_ (mT)	μ_0_ *H*_k_ (mT)
Co_40_Fe_40_B_20_/Pt_100.0_Bi_0.0_	0.0140 ± 0.0005	0.2877 ± 0.2530	1141.38 ± 4.36	12.17
Co_40_Fe_40_B_20_/Pt_96.1_Bi_3.9_	0.0200 ± 0.0007	0.5675 ± 0.3465	1268.79 ± 0.83	5.12
Co_40_Fe_40_B_20_/Pt_94.0_Bi_6.0_	0.0230 ± 0.0010	0.2690 ± 0.6140	1268.43 ± 7.22	9.95
Co_40_Fe_40_B_20_/Pt_91.3_Bi_8.7_	0.0290 ± 0.0020	0.1054 ± 0.9418	1088.84 ± 3.48	4.69
Co_40_Fe_40_B_20_	0.0120 ± 0.0004	0.1760 ± 0.2260	1038.91 ± 1.51	2.75

a Here, Δ*H*_0_ represents the inhomogeneous linewidth broadening obtained from the linear fit intercept in Figure 2d.

To quantify θ_SH_, we use DC-bias ST-FMR,^16^ where a direct current *I*_dc_ is applied simultaneously with *I*_rf_ under an external in-plane magnetic field *H*_ext_ to modulate the linewidth Δ*H*. Figure 3a–d shows the change in linewidth, δ­(μ_0_Δ*H*), as a function of *I*_dc_ at *f* = 8 GHz, measured at an angle ϕ = 70° with an input power of 4 dBm. Here, ϕ is the angle between the combined current direction (*I*_rf_ + *I*_dc_) and the external field *H*_ext_. The angle ϕ = 70° was chosen to maximize the linewidth modulation since sin ϕ reaches near its peak between 70° and 75°, and the AMR also exhibits significant variation within the 45°–75° range^18,63^ fruitful for a clean ST-FMR spectrum. The change in linewidth is defined as $\delta \left(\mu_{0}\Delta H\right)={\mu_{0}\Delta H|}_{I_{\mathrm{dc}}}-{\mu_{0}\Delta H|}_{I_{\mathrm{dc}}=0}$ , which varies linearly with *I*_dc_. Reversing the polarity of *H*_ext_ from positive *H*_ext_ at ϕ = 70° to negative *H*_ext_ at ϕ = 250° reverses the magnetization direction of Co_40_Fe_40_B_20_, which correspondingly reverses the slope of δ­(μ_0_Δ*H*)/*I*_dc_, consistent with the conventional SHE-driven SOT mechanism.^36^ Using a parallel-resistor model (see Section S5 for details on calculating ρ_*xx*_) and Figure S5 for ρ_*x* *x*_ values, we estimate the current density in the HM layer, *j*_dc,HM_, and extract θ_SH_ from the slope $\frac{\Delta [\delta (\mu_{0}\Delta H)]}{\Delta j_{\mathrm{dc},\mathrm{HM}}}$ according to^16^

$$\theta_{\mathrm{SH}}=\frac{2e}{\hbar }\frac{(H_{R}+\frac{M_{\mathrm{eff}}}{2})\mu_{0}M_{s}t}{\sin\phi }\frac{\gamma }{2\pi f}\left|\frac{\Delta [\delta (\mu_{0}\Delta H)]}{\Delta j_{\mathrm{dc},\mathrm{HM}}}\right|$$ 4

where *M*_s_ and *t* are the saturation magnetization and thickness of Co_40_Fe_40_B_20_, respectively, and *M*_eff_ is the effective demagnetization obtained from Kittel fitting.^61,64^ We observe a slight increase in θ_SH_ from 0.07 ± 0.01 (Pt_100.0_Bi_0.0_) to 0.12 ± 0.02 (Pt_96.1_Bi_3.9_), with a small doping of 3.9%. However, increasing the Bi concentration to 6.0% and 8.7% yields a remarkable enhancement in θ_SH_ to 0.24 ± 0.02 (Pt_94.0_Bi_6.0_) and 0.19 ± 0.01 (Pt_91.3_Bi_8.7_). In particular, the highest θ_SH_ occurs at higher Bi concentrations where Pt loses its crystalline texture, as seen in Figure 1. This supports the correlation between reduced crystallinity and enhanced θ_SH_ in 5*d* transition metals. Recent experimental studies by Parkin et al.^40^ and Shashank et al.^36^ have similarly demonstrated that θ_SH_ peaks near the crystalline–amorphous boundary, where extrinsic scattering from impurities is maximized.

**Figure 3:**
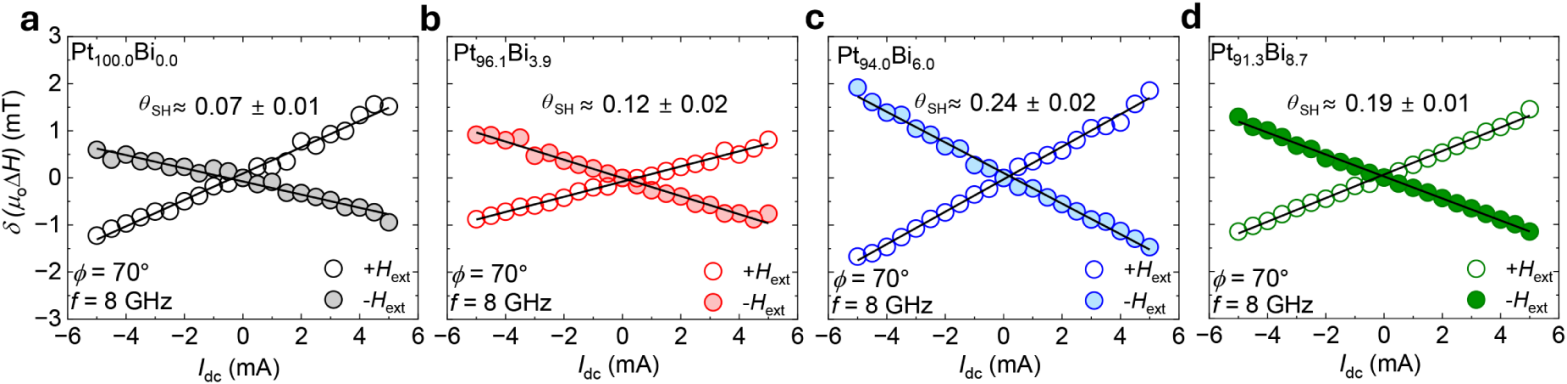
Current-induced modulation of linewidth, δ­(μ_0_Δ*H*) as a function of *I*_dc_ at *f* = 8 GHz for (a) Pt_100.0_Bi_0.0_, (b) Pt_96.1_Bi_3.9_, (c) Pt_94.0_Bi_6.0_, and (d) Pt_91.3_Bi_8.7_. Open circles correspond to positive magnetic field sweep (+*H*_ext_), while filled circles correspond to negative sweep (−*H*_ext_). The solid black lines are linear fits. The increasing slope of δ­(μ_0_Δ*H*)/*I*_dc_ with Bi impurity in Pt indicates enhanced θ_SH_.

### Spin Hall Nano-Oscillators

We fabricated 100 nm wide SHNOs and measured AO on all Co_40_Fe_40_B_20_/Pt_100–*x*_Bi_*x*_ heterostructures to observe the effect of a higher θ_SH_ on *I*_th_. See Experimental Section for details. A schematic of the setup is shown in Figure 4a. A direct current, *I*_dc_ (along the *x*-axis), was applied through a bias tee, while the resulting microwave signals were extracted through the high-frequency port, amplified using a low-noise amplifier (LNA), and analyzed with a spectrum analyzer (SA). A moderate out-of-plane field (θ = 84°) was selected to achieve a weak negative nonlinearity.^65^ The in-plane angle, ϕ = 20°, was chosen to ensure adequate electrical sensitivity of the signal (ϕ is the angle between the *y*-axis and *H*_ext_).

**Figure 4:**
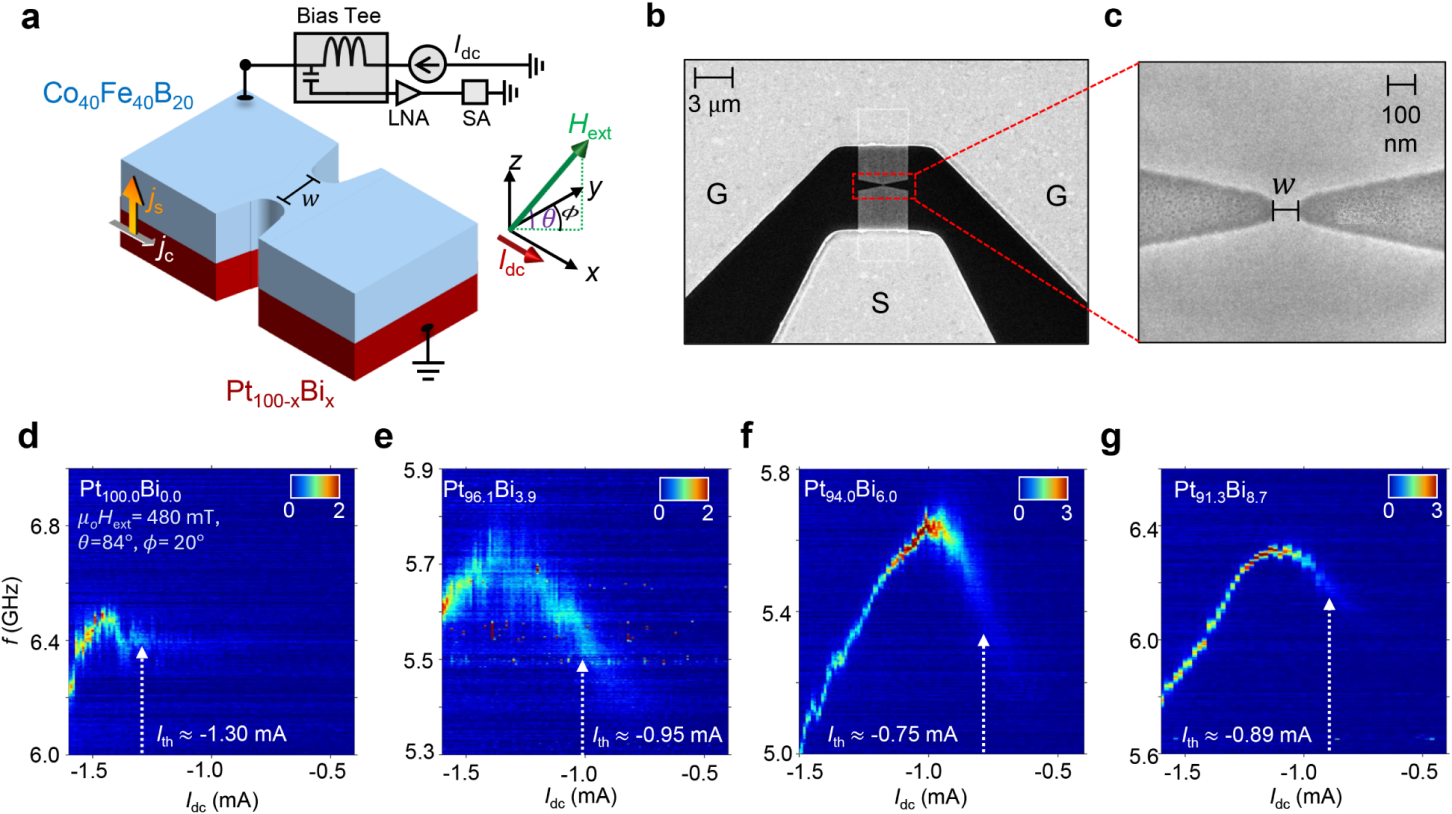
(a) Schematic of the SHNO and measurement setup. (b) SEM image of the GSG-based CPW with SHNO. (c) Enlarged SEM of the 100 nm wide SHNO (black scale bars in b, c). PSD from the 100 nm SHNO as a function of *I*_dc_ at μ_0_ *H*_ext_ = 480 mT, ϕ = 20°, θ = 84° for (d) Pt_100.0_Bi_0.0_, (e) Pt_96.1_Bi_3.9_, (f) Pt_94.0_Bi_6.0_, and (g) Pt_91.3_Bi_8.7_. Color bars indicate peak power (dB above noise floor). The white arrows mark the respective *I*_th_.

Figure 4b shows the scanning electron microscope (SEM) image of the device under test, and Figure 4c presents the enlarged SEM image of the 100 nm wide SHNO. The actual measured width *w* is in good agreement with the nominal design value. To understand the reciprocal relationship between the enhancement in θ_SH_ and the reduction in *I*_th_, we performed current-dependent AO and extracted the power spectral density (PSD) generated by the 100 nm wide SHNO under μ_0_ *H*_ext_ = 480 mT (ϕ = 20° and θ = 84°) for all samples, as shown in Figure 4d–g. A nonmonotonic current dependence of the AO frequency is observed, attributed to a localized AO bullet mode promoted by negative nonlinearity, N , typical for a FM layer with its magnetization far from the film normal.^65^ The *I*_th_ is −1.30 mA for Pt_100.0_Bi_0.0_, decreasing to −0.95 mA for Pt_96.1_Bi_3.9_. At higher Bi concentrations, *I*_th_ drops significantly to −0.75 mA (42% reduction) for Pt_94.0_Bi_6.0_ and −0.89 mA (32% reduction) for Pt_91.3_Bi_8.7_. Concurrently, the PSD maps in Figure 4d–g show that Pt_94.0_Bi_6.0_ and Pt_91.3_Bi_8.7_ reach peak powers of ∼3 dB above the noise floor, clearly exceeding the ∼2 dB observed for Pt_100.0_Bi_0.0_ and Pt_96.1_Bi_3.9_. The integrated spectral power is likewise enhanced, while the linewidth is reduced, indicating stronger and more coherent auto-oscillations at higher Bi concentrations, which is advantageous for wireless communication applications.^24^ Additional auto-oscillation measurements performed at μ_0_ *H*_ext_ = 520–680 mT (see Figure S7) show that the extracted *I*_th_ exhibits only weak dependence on the applied magnetic field within this highly out-of-plane field geometry, consistent with previous reports on nanoconstriction SHNOs where the threshold current is governed by the interplay between the Gilbert damping parameter α_total_, internal field, spin-wave localization, and spin–orbit torque.^66^ See Figure S6 for AMR, Figure S7 for AO at other fields, Figure S8 for *I*_th_ extraction, and Figure S9 for peak power, output power, and linewidth.

### Reciprocity, Interfacial Transparency, and Mechanism of Spin Hall Effect in PtBi Alloys

Building on the structural analysis and the observed reciprocity between ST-FMR and AO, several key parameters, ρ_*xx*_, *T*_int_, θ_SH_ (including *T*_int_), and *I*_th_, are analyzed across all Pt_100–*x*_Bi_*x*_ alloys (see Figures 5a–c, S9 and S10). The electrical resistivity ρ_*xx*_ increases with Bi concentration, rising from 65 μΩ·cm for 0.0% Bi to 270, 288, and 301 μΩ·cm for 3.9%, 6.0%, and 8.7% Bi, respectively (Figure 5a). To account for interfacial transparency effects, we evaluated the interfacial spin transparency *T*_int_ following the standard method by Parkin et al.^32^ *T*_int_ was determined using the effective spin mixing conductance^35^ $g_{\mathrm{eff}}^{↑↓}$ obtained from linewidth broadening, the real part of the spin mixing conductance *G* ^↑↓^, and the spin diffusion length λ_sd_ ∝ 1/ρ_*xx*_ (Elliott–Yafet mechanism). The resulting *T*_int_ values gradually increase with Bi concentration, reflecting improved spin-current transmission across the Co_40_Fe_40_B_20_/Pt_100–*x*_Bi_*x*_ interface (see Figure S10). When normalizing θ_SH_ by *T*_int_, the overall trend remains unchanged: θ_SH_/*T*_int_ increases up to 6% Bi and saturates beyond this concentration, confirming that the primary enhancement originates from bulk PtBi alloying rather than interface effects. The reported θ_SH_ values in the main text thus represent lower bounds, while θ_SH_/*T*_int_ provides an interface-corrected estimate (see Figure 5b). For 0.0% and 3.9% Bi doping, θ_SH_ increases from 0.07 to 0.12, with a corresponding rise in ρ_*xx*_ from 65 to 270 μΩ·cm, reducing *I*_th_ from −1.30 mA to −0.97 mA. At 6.0% Bi, ρ_*xx*_ rises marginally to 288 μΩ·cm, while θ_SH_ significantly rises to 0.24 (3.4× increase), yielding the lowest *I*_th_ of −0.75 mA (42% reduction). At 8.7% Bi, ρ_*xx*_ further increases to 301 μΩ·cm, but θ_SH_ decreases slightly to 0.19, still 2.7× higher than 0.0% Bi (Pt_100.0_Bi_0.0_), causing *I*_th_ to slightly rise to −0.89 mA, but still 32% lower than 0.0% Bi. Importantly, the 3.4× and 2.7× enhancement in θ_SH_ directly corresponds to the 42% and 32% reduction in *I*_th_, respectively, establishing a clear correlation between θ_SH_ enhancement and *I*_th_ suppression, as shown in Figure 5b,c. This correspondence strengthens the conclusion that 6–8.7% Bi marks the critical range governing both microstructural evolution and spin transport behavior in PtBi alloys. Notably, the spin pumping study^44^ also reports that θ_SH_ peaks within the 6–8% Bi range, consistent with the critical compositional window identified in our work. Despite the increase in α_total_ with Bi doping, the enhanced **τ**_DL_ from the SHE compensates for **τ**_α_, leading to a reduction in *I*_th_. This reduction would likely have been more significant if α_total_ had remained constant or decreased, complicating the direct assessment of SOT effects. The actual effect of torque on the AO is thus likely underestimated, and FM layers with lower α_total_, such as GdFeCo,^61^ could further enhance the reduction in *I*_th_. Furthermore, this interplay between θ_SH_ and ρ_*xx*_ is critical for minimizing power consumption, expressed by the factor $\rho_{\mathrm{xx}}/\theta_{\mathrm{SH}}^{2}$ relevant for SOT-MRAM applications, which improves at higher Bi concentrations (6.0% and 8.7%).^36,40^

**Figure 5:**
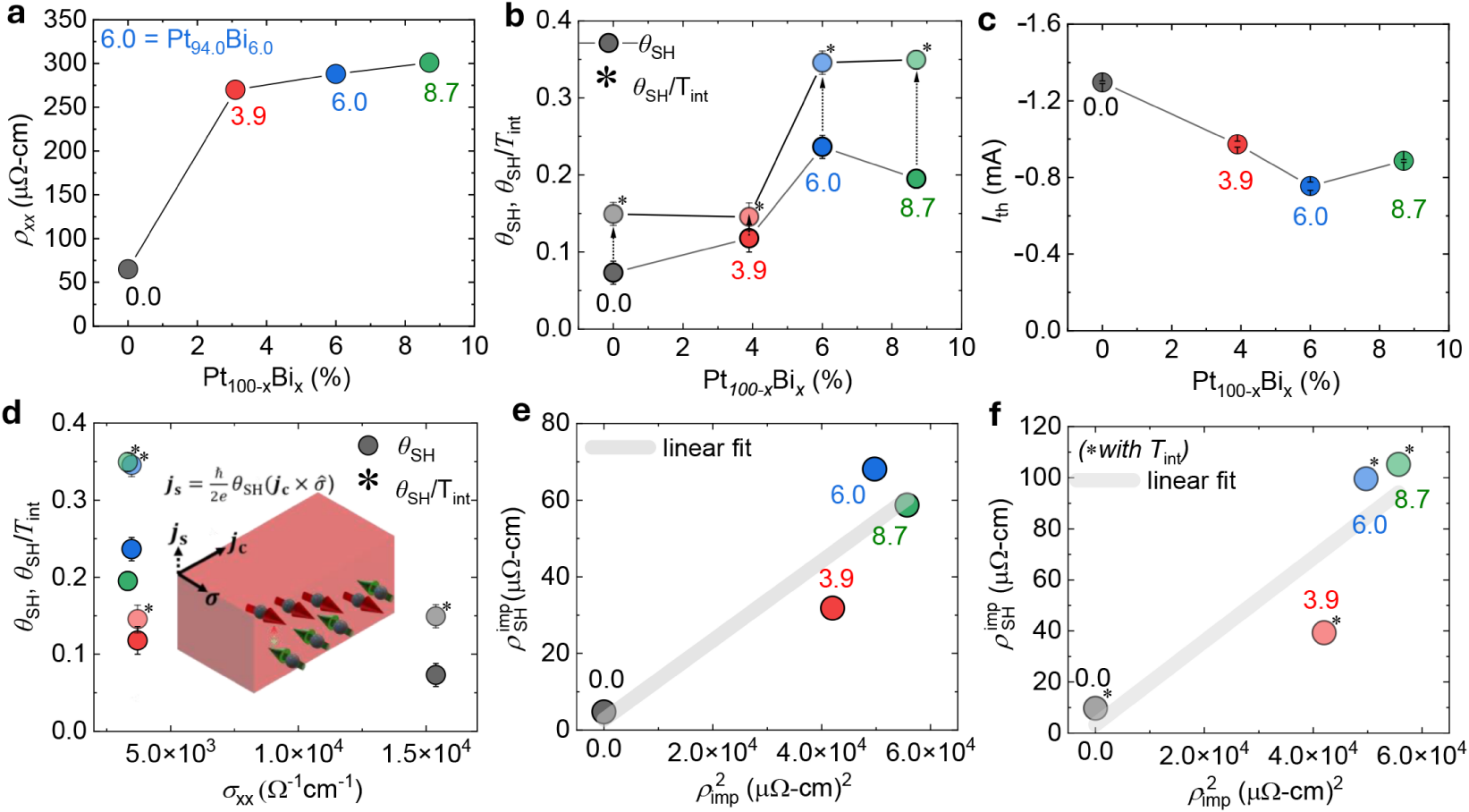
(a, b) Electrical resistivity, ρ*_xx_*, θ_SH_ (solid circles), and interface-corrected θ_SH_/*T*_int_ (translucent circles) versus Bi content *x* in Pt_100–*x*_Bi*_x_* (at.%). (c) *I*_th_ versus Bi content. (d) θ_SH_ and θ_SH_/*T*_int_ plotted against σ*_xx_*; inset shows schematic of bulk spin Hall effect in PtBi alloy. (e) Impurity-induced spin Hall resistivity $\rho_{\mathrm{imp}}^{\mathrm{SH}}$ versus impurity resistivity squared $\rho_{\mathrm{imp}}^{2}$ (without transparency correction). (f) Same plot as (e) but corrected using *T*_int_, showing that the linear scaling $\rho_{\mathrm{imp}}^{\mathrm{SH}}\propto \rho_{\mathrm{imp}}^{2}$ persists. Error bars in (b, d) represent the standard error from eqs 2 and 4; in (c), mean deviation (MD) from *I*_th_ data sets (see Figure S7 and Experimental Section for details).

To elucidate the mechanism underlying the SHE, we examine electrical conductivity σ_*xx*_. Figure 5d shows θ_SH_ and θ_SH_/*T*_int_ *vs σ*_*xx*_, with values lying in the bad-metal/dirty-metal regime (σ_*xx*_ ≲ 10^4^ Ω^–1^ cm^–1^). In this regime, a linear dependence of θ_SH_ on σ_*xx*_ indicates a dominant intrinsic SHE contribution in Pt, as demonstrated by Casanova et al.^67^ However, no clear linear trend is observed, suggesting coexistence of intrinsic and extrinsic side-jump scattering mechanisms^36,55,67^ (see Figure S11 and Section S12 of the Supporting Information). Moreover, the increase of θ_SH_ and θ_SH_/*T*_int_ with ρ_*xx*_ supports a dominant role of side-jump scattering and/or disorder-enhanced intrinsic contributions.^39^ In metals with strong spin–orbit coupling, the anomalous Hall effect scales with ρ_*xx*_ either linearly (extrinsic skew scattering) or quadratically (extrinsic side-jump scattering or intrinsic mechanisms).^68^ Recent studies have established that SHE shares this scaling behavior.^68^ The observed quadratic dependence of θ_SH_ or θ_SH_/*T*_int_ on ρ_*xx*_ hints at the contribution of bulk-extrinsic side-jump scattering and rules out significant interfacial Bi effects on SHE, as confirmed by TEM and consistent with bulk alloy characteristics.

To further confirm the mechanism, the intrinsic SHE contribution was subtracted, revealing $\rho_{\mathrm{imp}}^{\mathrm{SH}}\propto \rho_{\mathrm{imp}}^{2}$ , with and without considering *T*_int_ (Figure 5e,f), indicative of extrinsic side-jump scattering dominance.^34,35,41,55,62^ Here, $\rho_{\mathrm{imp}}^{\mathrm{SH}}$ denotes the impurity-induced spin Hall resistivity, and ρ_imp_ the resistivity due to impurities. Therefore, increasing Bi concentration in Pt enhances extrinsic side-jump scattering, substantially boosting θ_SH_ and reducing the threshold current *I*_th_ necessary to drive robust AO, thereby enabling energy-efficient SHNOs.

### Performance Enhancement in PtBi Alloy SHNOs

The resistivity ρ_*xx*_ of Pt_100–*x*_Bi_*x*_ alloys strongly influences SHNO performance and energy efficiency through current redistribution in the nanoconstriction. Together with a modest increase in the AMR ratio at higher Bi concentrations (6.0% and 8.7%; Figures S3 and S6), this leads to enhanced microwave output power. To benchmark SHNO performance for wireless communication applications, we evaluate the quality factor *Q*, defined as *Q* = *f*/Δ*f*.^21,22,24^ As shown in Figure 6a, SHNOs with higher Bi content exhibit reduced linewidths of ≈25 MHz and increased auto-oscillation frequencies, resulting in high quality factors of *Q* ≈ 350–550 within the normalized current range *I*_dc_/*I*_th_ = 1.1–1.6. These values correspond to an approximately 4-fold enhancement compared to 0.0% Bi devices.

**Figure 6:**
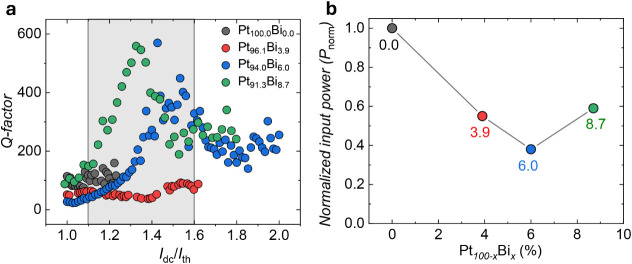
(a) *Q* factor for 100 nm wide SHNOs with varying Bi content in Pt_100–*x*_Bi*_x_* alloys as a function of *I*_dc_/*I*_th_. The values are normalized with the respective *I*_th_. Data are extracted from Figures 4d–g and S9a–d. The shaded gray region indicates the normalized current range *I*_dc_/*I*_th_ = 1.1–1.6 used for comparison. The figure highlights the performance enhancement due to Bi doping, showing the reduction in linewidth and threshold current, as well as the improvement in output power and *Q* factor, particularly at higher Bi concentrations. (b) Normalized input power for 100 nm wide SHNOs, showing a remarkable 61.6% and 41.0% reduction for 6.0% and 8.7% Bi, respectively.

The effective power consumption of SHNOs is given by $P=I_{\mathrm{th}}^{2}R$ , where *I*_th_ is the threshold current and *R* is the total device resistance.^49^ The normalized input power, *P*_norm_ = *P*/*P*_0_ (with *P*_0_ corresponding to 0.0% Bi (Pt_100.0_Bi_0.0_)), is shown in Figure 6b as a function of Bi concentration. Bi alloying leads to a pronounced reduction in power consumption, reaching a minimum *P*_norm_ = 0.38 at 6.0% Bi, corresponding to a 61.6% reduction relative to 0.0% Bi. At higher Bi content (8.7%), *P*_norm_ slightly increases to 0.59 (41.0% reduction), indicating that optimal energy efficiency is achieved at moderate Bi concentrations. This reduction in power consumption is driven by the lowered *I*_th_ resulting from enhanced spin–orbit torque in PtBi alloys and is comparable to the maximum reductions reported for W–Ta alloy-based SHNOs, where reduced crystallinity/disorder can coexist with enhanced spintronic functionality and enhanced SOT/SHE.^49^ Unlike van der Waals (vdW) heterostructures, where high crystalline quality is often essential for spintronic functionality,^69^ moderately reduced crystallinity and alloy disorder in PtBi can be beneficial for enhancing the SHE through extrinsic scattering mechanisms.^36^ Overall, these results link Bi-induced crystallographic disorder and enhanced θ_SH_ to reduced *I*_th_, increased *Q* factor, and lower power consumption, underscoring the effectiveness of Bi doping for energy-efficient Pt-based SHNOs.

## Conclusion

In summary, we demonstrate that Bi doping in Pt enhances θ_SH_ by over 3-fold and substantially reduces the threshold current *I*_th_ in SHNOs, enabling a superior ρ_*xx*_ trade-off crucial for low-power spintronic applications. The concurrent enhancement of θ_SH_ and reduction in *I*_th_ underscores the reciprocal relationship between the spin Hall effect and auto-oscillations, as revealed by complementary ST-FMR and AO measurements. Due to a lower Δ*f* and higher auto-oscillation frequency (*f*) at higher Bi concentrations of 6.0% and 8.7%, we obtain a *Q* factor of ≈ 350 ≤ *Q* ≤ 550 attained within the *I*_dc_/*I*_th_ range of 1.1–1.6, which is approximately four times higher than that of 0.0% Bi (Pt_100.0_Bi_0.0_). Due to a reduction in *I*_th_, we report a 61.6% reduction in power consumption underlying the performance enhancement in SHNOs for energy-efficient operation. Furthermore, in-plane angular-dependent ST-FMR confirms a sin 2ϕ cos ϕ symmetry across all Pt_100–*x*_Bi_*x*_ alloys, indicative of a bulk-origin, conventional spin–orbit torque mechanism. GIXRD analysis and diffractograms from HR-TEM confirm that increased Bi doping in Pt leads to progressive reduction in crystallinity, significant peak broadening, and preferred crystallographic orientation, indicating structural modifications in the Pt_100–*x*_Bi_*x*_ alloys. These findings are corroborated by cross-sectional TEM and STEM-EDXS intensity line profiles, which reveal a uniform distribution of Bi throughout the Pt layer and a concurrent loss of Pt crystallinity. The nonlinear dependence of θ_SH_ on σ_*xx*_ and ρ_*xx*_ further reveals a $\rho_{\mathrm{imp}}^{\mathrm{SH}}\propto \rho_{\mathrm{imp}}^{2}$ scaling, with and without the *T*_int_ factor, confirming extrinsic side-jump scattering as the dominant mechanism behind the enhanced SHE. Collectively, our findings establish a framework for energy-efficient spintronics beyond conventional 5*d* transition metals. By demonstrating Bi alloying with Pt as an effective strategy for enhancing charge-to-spin conversion, our work offers design principles for next-generation SHNOs for applications in neuromorphic computing, SOT-MRAM, Ising machines, GHz spintronic devices, and wireless communication.

## Experimental Section

### Thin Film Preparation

High-resistance silicon (HR-Si) wafers (>10,000 Ω·cm) were used as substrates. The Pt_100–*x*_Bi_*x*_(4.0 nm)/Co_40_Fe_40_B_20_(3.0 nm) stacks were grown on HR-Si/Ta­(2.4 nm). Control samples, Co_40_Fe_40_B_20_(3.0 nm) and Ta­(2.4 nm) were grown on HR-Si. All samples were further protected with a capping layer of MgO­(2.0 nm)/SiO_2_(3.0 nm). Ta and Co_40_Fe_40_B_20_ were sputtered at 11 W with 13.90 ± 0.06 sccm Ar flow at a base pressure of 4 × 10^–8^ mbar, rising to 4 × 10^–3^ mbar during deposition. Other layers were deposited via electron beam evaporation. Pt_100–*x*_Bi_*x*_ was synthesized *in situ* by coevaporation, with deposition rates monitored using a quartz crystal microbalance (QCM). The Bi source was stabilized before Pt adjustment. The deposition rate ratio required to achieve the desired Pt_100–*x*_Bi_*x*_ composition was calculated as $\frac{v_{a}}{v_{b}}=\frac{M_{a}\rho_{b}}{M_{b}\rho_{a}}\cdot r$ , where *v*_a_ and *v*_b_ are the deposition rates, *M*_a_ and *M*_b_ are the molar masses, ρ_a_ and ρ_b_ are the material densities, and *r* is the target molar ratio.^51^

### Grazing-Incidence X-ray Diffraction (GIXRD)

GIXRD measurements were used to determine the full-width-at-half-maximum (FWHM) of the diffraction peaks. Data were acquired using a Mat:Nordic SAXSLAB instrument with a Rigaku 003 microfocusing Cu X-ray source (parallel beam from a two-bounce monochromator) and two Dectris detectors: Pilatus3 300KR (orthogonal) and 100K (goniometer circle). The beam path was evacuated to suppress air scattering, and a 1° incidence angle was employed to maximize signal intensity. Two-dimensional diffraction images were processed using SAXSGUI. FWHM values and lattice parameters were determined using TOPAS v6 (Bruker AXS, 2016), applying a pure platinum (Fm3̅m) structural model and a pseudo-Voigt (PVII) profile. Peak broadening was analyzed separately. Exposure time was 3 h per sample. Instrumental broadening was assessed using LaB_6_ powder.

### Cross-Sectional TEM Imaging and STEM-EDXS

TEM measurements were carried out using a JEOL monochromatic ARM200F microscope, which is equipped with a Schottky field emission gun, a double-Wien monochromator, a probe aberration corrector, an image aberration corrector, a Gatan image filter (GIF) Continuum, a Gatan OneView camera, as well as a double silicon drift detector (SDD) for energy-dispersive X-ray spectroscopy (EDXS). The microscope was operated at 200 kV for the TEM measurements. TEM specimens were prepared using an FEI Versa 3D focused ion beam-scanning electron microscope (FIB-SEM). After depositing a protective layer containing Pt and C using first the electron beam and then the Ga ion beam in the FIB-SEM, a lamella of the material was cut out using an ion beam at 30 kV and 1 nA. After transferring the lamella to a Cu TEM grid, the lamella was gradually thinned down by the ion beam. The thinning process was performed first at 30 kV with a gradually decreasing beam current from 1 nA to 100 pA. Then, gentle polishing of the specimen was carried out with ion beam energies of 5 kV and 2 kV to minimize ion beam effects.

### Device Fabrication

ST-FMR bars (14 × 4 μm^2^) and SHNOs with a width of 100 nm were fabricated via electron-beam lithography (Raith EBPG 5200), followed by Ar-ion milling. The GSG-CPW contact pads were subsequently fabricated by mask-less ultraviolet lithography (Heidelberg Instruments MLA 150) and a liftoff technique, followed by Cu­(800 nm)/Pt­(20 nm) electrodes deposited by DC magnetron sputtering. The detailed fabrication process can be found in ref 70.

### AMR, ST-FMR, and AO Measurements

The AMR of all SHNOs and ST-FMR bars was extracted from two-point resistance measurements performed with a rotatable in-plane magnetic field using a Keithley 2182A nanovoltmeter and a Keithley 6221 DC and AC current source. ST-FMR measurements were conducted with a PhaseFMR-40ST instrument from NanOsc Instruments AB. For angular ST-FMR measurements, the sample was measured under a rotatable in-plane magnetic field using a Rohde and Schwarz SMB100A signal generator and a Stanford Research Systems SR830 lock-in amplifier to generate *I*_rf_ and detect the ST-FMR voltage *V*_dc_, respectively. For AO measurements, *I*_dc_ was applied using a Keithley 2400 sourcemeter, and the AO signal was amplified using a Low Noise Factory (LNF) LNR4 14B low-noise amplifier and detected using a Rohde and Schwarz FSV spectrum analyzer with a resolution bandwidth of 1 MHz. All measurements were performed at room temperature.

### Statistical Analysis

The mean deviation (MD) quantifies dispersion in a data set *x*_1_, *x*_2_, ..., *x*_*n*_ and is computed with respect to the mean (*x̅*) or median (*M*). The mean deviation about the mean is

$$MD_{\mathrm{x̅}}=\frac{1}{n}\overset{n}{\underset{i=1}{\sum }}\left|x_{i}-\mathrm{x̅}\right|$$

where *x̅* is the arithmetic mean, $\mathrm{x̅}=\frac{1}{n}\overset{n}{\underset{i=1}{\sum }}x_{i}$ . Here, |*x*_*i*_ – *x̅*| represents the absolute deviation from the mean.

## Supplementary Material



## Data Availability

The data that support the findings of this study are available from the corresponding authors upon reasonable request.

## Notes

HAADF-STEM and STEM-EDXS characterization; angular-dependent AMR and ST-FMR measurements; power consumption analysis; auto-oscillation measurements at additional external magnetic fields; threshold current extraction; spin Hall nano-oscillator performance analysis; spin mixing conductance and interfacial spin transparency analysis; spin Hall performance comparison of Pt-based alloys; and spin Hall effect mechanism (PDF)
