# Supplementary material for: Bulk Spin–Orbit Torque-Driven Spin Hall Nano-Oscillators Using PtBi Alloys with Engineered Crystallinity
Source: ACS Appl Mater Interfaces. 2026 Jul 13;18(29):39941–51. doi: 10.1021/acsami.6c06092 (PMC13425562; doi:10.1021/acsami.6c06092)
Supplement: Supplementary file 1 [file am6c06092_si_001.pdf]

Supporting Information

Bulk spin-orbit torque-driven spin Hall nano-oscillators using PtBi alloys with engineered crystallinity

Utkarsh Shashank,[†] Akash Kumar,[†,‡,¶] Tahereh Sadat Parvini,[§,‖,⊥] Hauke Heyen,[§] Lunjie Zeng,[#] Andrew B. Yankovich,[#] Jong-Guk Choi,[†] Mona Rajabali,[@] Eva Olsson,[#] Markus Münzenberg,[*,§] and Johan Åkerman[*,†,‡,¶]

†*Applied Spintronics Group, Department of Physics, University of Gothenburg, 412 96 Gothenburg, Sweden.*

‡*Center for Science and Innovation in Spintronics, Tohoku University, 2-1-1 Katahira, Aoba-ku, Sendai 980-8577 Japan*

¶*Research Institute of Electrical Communication, Tohoku University, 2-1-1 Katahira, Aoba-ku, Sendai 980-8577 Japan*

§*Institut für Physik, Universität Greifswald, Greifswald 17489, Germany*

‖*Walther-Meißner-Institut, Bayerische Akademie der Wissenschaften, 85748 Garching, Germany*

⊥*Munich Center for Quantum Science and Technology (MCQST), Schellingstr.4, 80799, Munich, Germany*

#*Department of Physics and Astronomy, Chalmers University of Technology, 412 96 Gothenburg, Sweden*

@*NanOsc AB, Kista, Sweden.*

E-mail: markus.muenzenberg@uni-greifswald.de; johan.akerman@physics.gu.se

Table of contents

S1: HAADF-STEM and STEM-EDXS data for $Pt_{100.0}Bi_{0.0}$ stack

S2: HAADF-STEM and STEM-EDXS data/line profile for $Pt_{94.0}Bi_{6.0}$ and $Pt_{91.3}Bi_{8.7}$

S3: In-plane angular AMR measurements for ST-FMR micro-strips

S4: Angular dependence of SOT via in-plane angular ST-FMR measurements

S5: Power consumption ratio in SOT-MRAM

S6: In-plane angular AMR measurements for 100 nm wide SHNO

S7: Auto-Oscillation at other fields for 100 nm wide SHNO

S8: Extraction of threshold current

S9: Impact of resistivity, ρ_{xx} of $Pt_{100-x}Bi_x$ alloys on SHNO performance

S10: Spin mixing conductance, interfacial spin transparency, and their impact on θ_{SH}

S11: Spin Hall performance of Pt-based alloys

S12: Mechanism of SHE

S1: HAADF-STEM and STEM-EDXS elemental maps for $Pt_{100.0}Bi_{0.0}$ stack

Figure S1 shows the HAADF-STEM image and STEM-EDXS elemental maps of the $Pt_{100.0}Bi_{0.0}$ stack.

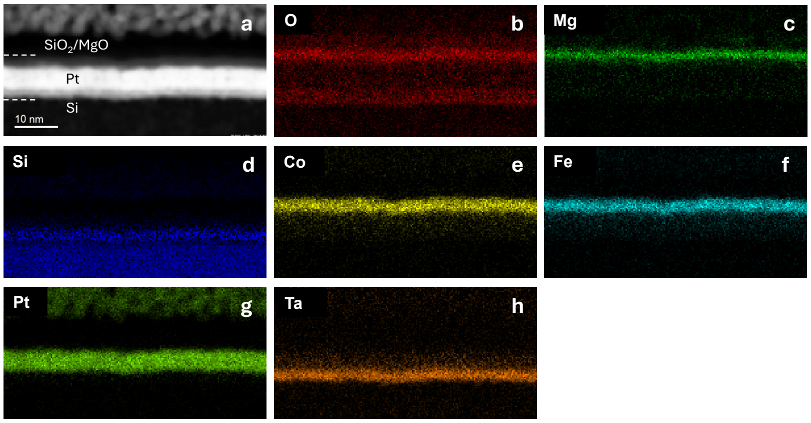

Figure S1. a)–h) STEM-EDXS data from $Pt_{100.0}Bi_{0.0}$ Stack (HR-Si/Ta/Pt/$Co_{40}Fe_{40}B_{20}$/MgO/SiO_2). HAADF-STEM image of a) $Pt_{100.0}Bi_{0.0}$ stack. EDXS elemental maps of b) O, c) Mg, d) Si, e) Co, f) Fe, g) Pt, and h) Ta, represented by red, green, royal blue, yellow, turquoise, fluorescent green, and orange respectively.

S2: HAADF-STEM and STEM-EDXS elemental maps/line profile for $Pt_{94.0}Bi_{6.0}$ and $Pt_{91.3}Bi_{8.7}$

Figure S2 presents the HAADF-STEM images, STEM-EDXS elemental maps, and EDXS line profiles of the $Pt_{94.0}Bi_{6.0}$ and $Pt_{91.3}Bi_{8.7}$ stacks.

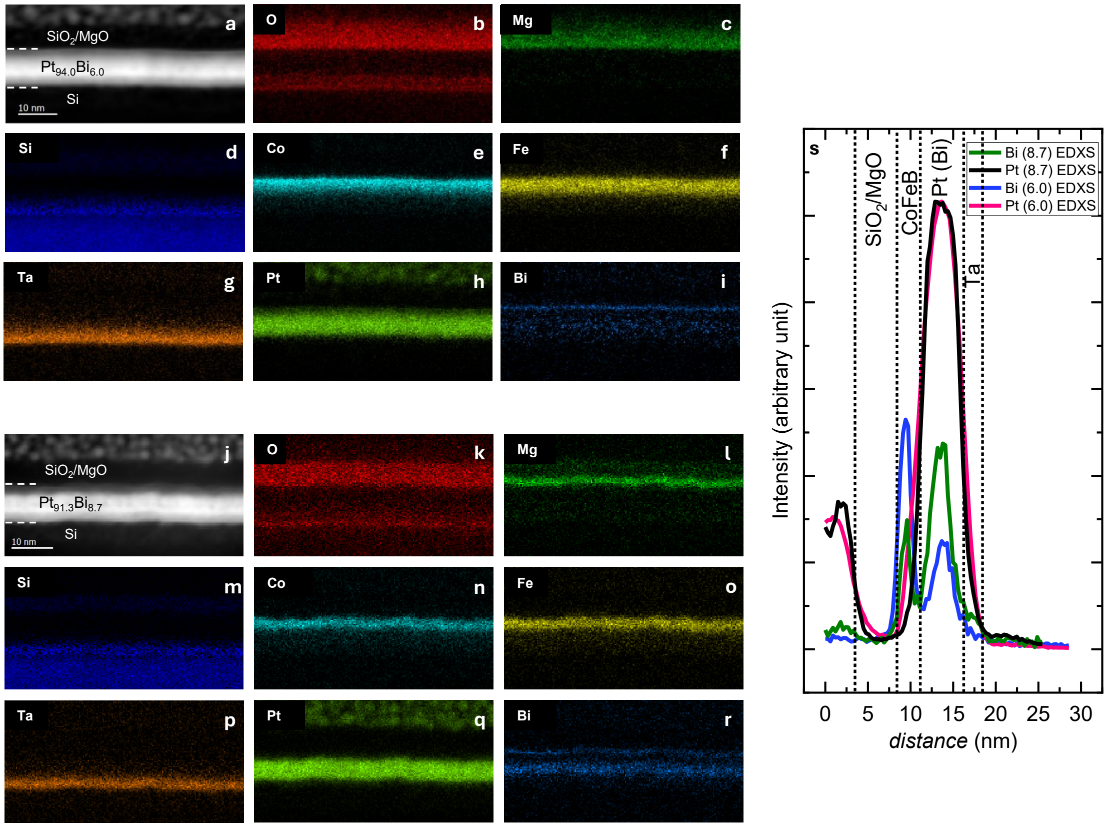

Figure S2. a)–i) STEM-EDXS data from the $Pt_{94.0}Bi_{6.0}$ stack (HR-Si/Ta/$Pt_{94.0}Bi_{6.0}$/$Co_{40}Fe_{40}B_{20}$/MgO/SiO_2). Panel a) is the HAADF-STEM image. Panels b)–i) show EDXS elemental maps for b) O, c) Mg, d) Si, e) Co, f) Fe, g) Ta, h) Pt, and i) Bi. Colors correspond to red, green, royal blue, turquoise, yellow, orange, fluorescent green, and navy blue, respectively. j)–r) STEM EDXS data from the $Pt_{91.3}Bi_{8.7}$ stack (HR-Si/Ta/$Pt_{91.3}Bi_{8.7}$/$Co_{40}Fe_{40}B_{20}$/MgO/SiO_2). Panel j) is the HAADF-STEM image. Panels k)–r) show EDXS elemental maps of k) O, l) Mg, m) Si, n) Co, o) Fe, p) Ta, q) Pt, and r) Bi, using the same color scheme as above. s) EDXS line profiles of Pt and Bi across the film thickness. Intensities are normalized to the Pt peak of each composition. Dashed lines mark the individual layers in the stacks, including the targeted Pt(Bi) layer, which shows no evidence of Bi clustering, confirming uniform elemental distribution and supporting the mechanism of bulk SHE in Bi doped Pt channel. The $Pt_{91.3}Bi_{8.7}$ sample displays a higher Bi signal within the Pt(Bi) layer than the $Pt_{94.0}Bi_{6.0}$ sample, consistent with higher nominal Bi content.

S3: In-plane angular AMR measurements for ST-FMR micro-strips

Figure S3a–c presents the in-plane angular anisotropic magnetoresistance (AMR) measurements for all ST-FMR microstrips at $\mu_0 H_{\text{ext}} = 100$, 150, and 200 mT. The AMR exhibits a $\cos^2(\phi)$ dependence, leading to an ST-FMR voltage rectification with $\Delta R \propto \sin(2\phi)$, confirming the absence of angular artifacts.

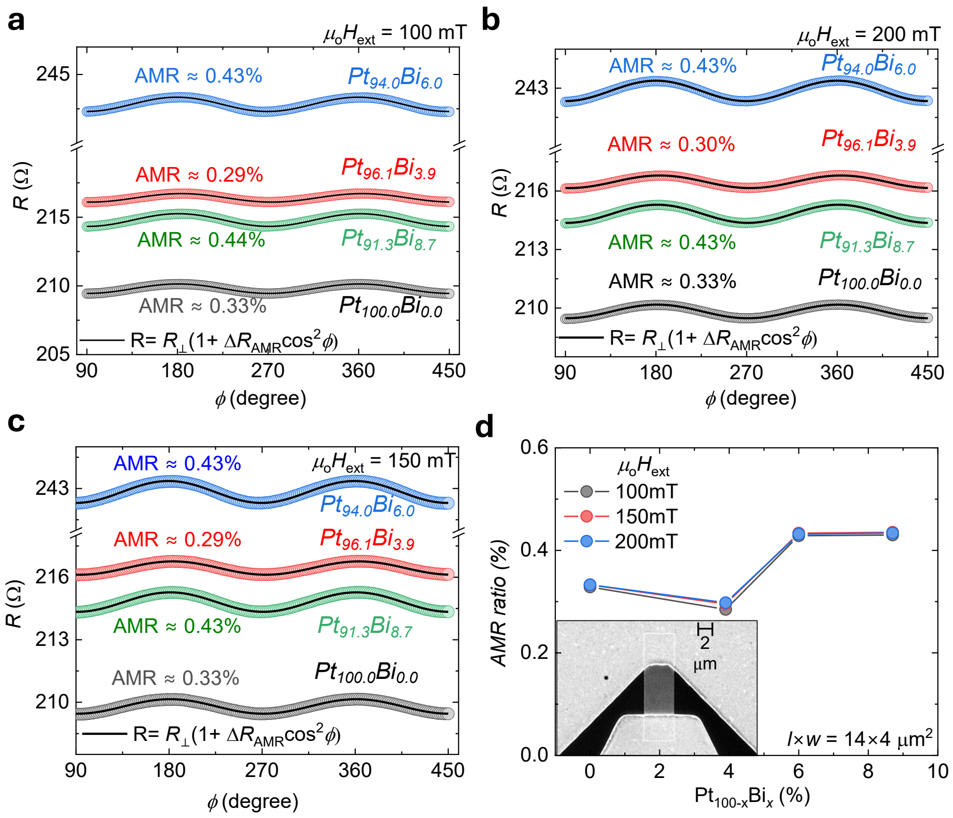

Figure S3. In-plane angular-dependent resistance measurements of micro-strips at a) 100 mT, b) 200 mT, and c) 150 mT for all $Pt_{100-x}Bi_x/Co_{40}Fe_{40}B_{20}$ hetero-structures, fitted using $R = R_\perp (1 + \Delta R_{\text{AMR}} \cos^2 \phi)$. Here, $R_\perp$ and $R_\parallel$ are the resistances at $\phi = 90°$ and $\phi = 180°$, respectively. ΔR_{AMR} is the relative change in resistance, given by $\frac{R_\parallel - R_\perp}{R_\perp}$. d) AMR ratio as a function of $Pt_{100-x}Bi_x$. The inset in d) shows the SEM image of a representative microstrip.

The AMR ratio is comparable for $Pt_{100.0}Bi_{0.0}$ (0.33%) and $Pt_{96.1}Bi_{3.9}$ (0.29%), increasing slightly to 0.43% for $Pt_{94.0}Bi_{6.0}$ and $Pt_{91.3}Bi_{8.7}$. This increase likely arises from higher ρ_{xx} in these samples, which redirects more current into the CoFeB layer (Figure S3d).

S4: Angular dependence of SOT via in-plane angular ST-FMR measurements

We utilized the in-plane angular ST-FMR measurements by varying ϕ between I_{rf} and H_{ext} from 0° to 360° for the $\mathrm{Co_{40}Fe_{40}B_{20}/Pt_{100-x}Bi_x}$ hetero-structures, measured at 8 GHz. For a given heavy metal/ferromagnet (HM/FM) system where the FM layer has in-plane magnetization, the damping-like torque ($\boldsymbol{\tau}_{\mathrm{DL}}$) and field-like torque ($\boldsymbol{\tau}_{\mathrm{OF}}$) can be either in-plane (IP) or out-of-plane (OOP), depending on the direction of the spin polarization vector ($\hat{\sigma}$) and the applied magnetic field (H_{ext}). The conventional damping-like torque ($\boldsymbol{\tau}_{\mathrm{DL},y} \propto \mathbf{m}\times(\hat{\boldsymbol{\sigma}}\times\mathbf{m}) \propto \cos\phi$) and the conventional Oersted field/field-like torque ($\boldsymbol{\tau}_{\mathrm{FL+Oe},y} \propto -(\hat{\boldsymbol{\sigma}} \times \mathbf{m}) \propto \cos\phi$) both exhibit a cosine dependence on ϕ. The ST-FMR voltage can be decomposed into the weight factors S and A, corresponding to the symmetric and antisymmetric part, respectively, when combined with the AMR detection of $\sin 2\phi$ can be expressed as:

$$\mathrm{S}(\phi) = \sin 2\phi\,(\tau_{\mathrm{DL},x}\sin\phi + \tau_{\mathrm{DL},y}\cos\phi + \tau_{\mathrm{FL},z}) \tag{A1}$$

$$\mathrm{A}(\phi) = \sin 2\phi\,(\tau_{\mathrm{FL},x}\sin\phi + \tau_{\mathrm{FL},y+\mathrm{Oe},y}\cos\phi + \tau_{\mathrm{DL},z}) \tag{A2}$$

Here, $\tau_{\mathrm{DL},y}$ is the conventional in-plane damping-like torque from the spin polarization along y. Whereas $\tau_{\mathrm{DL},x}$, and $\tau_{\mathrm{DL},z}$ denote the unconventional in-plane and out-of plane damping-like torque, arising from the spin polarization along x and z, respectively.[1] On the other hand, $\tau_{\mathrm{FL},z}$ and $\tau_{\mathrm{FL},x}$ is the unconventional in-plane and out-of-plane field-like torque, respectively. Whereas, $\tau_{\mathrm{FL},y+\mathrm{Oe},y}$ is the conventional out-of plane torque. Therefore, for a bulk-conventional SOT, both the weight factors S and A, must yield a $\sin 2\phi\cos\phi$. Figure S4a-d shows the angular dependent ST-FMR for $\mathrm{Pt_{100.0}Bi_{0.0}}$, $\mathrm{Pt_{96.1}Bi_{3.9}}$, $\mathrm{Pt_{94.0}Bi_{6.0}}$ and $\mathrm{Pt_{91.3}Bi_{8.7}}$, respectively for the weight factors S and A as a function of ϕ. Both the S and A are well fitted with the $\sin 2\phi\cos\phi$.

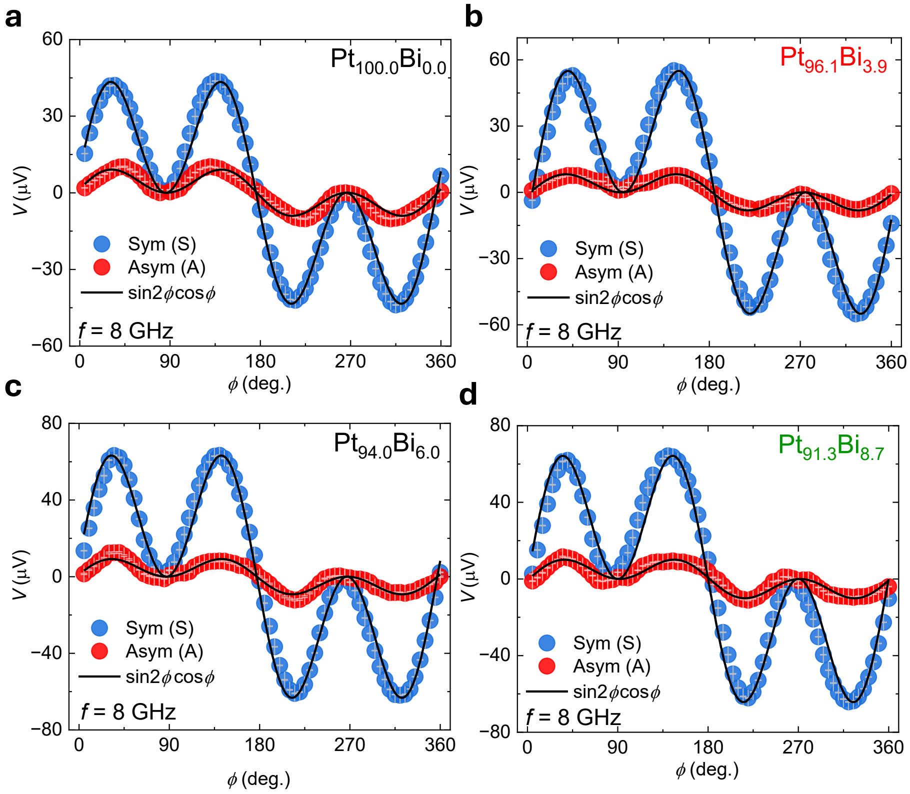

Figure S4. Weight factors S and A as a function of ϕ for a) $Pt_{100.0}Bi_{0.0}$, b) $Pt_{96.1}Bi_{3.9}$, c) $Pt_{94.0}Bi_{6.0}$, and d) $Pt_{91.3}Bi_{8.7}$, measured at 8 GHz. The solid black lines represent fits to the function $\sin 2\phi \cos \phi$.

S5: Power consumption ratio in SOT-MRAM

The power consumption in SOT-MRAM devices is influenced by the spin Hall material (SHM), which is $Pt_{100-x}Bi_x$ alloys here, and is known to scale as ρ_{xx}/θ_{SH}^2, assuming the adjacent ferromagnetic (FM) layer (here $Co_{40}Fe_{40}B_{20}$) remains unchanged, as shown by Liu *et al.*[2] This is because the critical current density required for magnetization switching is inversely proportional to θ_{SH}. To achieve lower power consumption, it is therefore important to enhance θ_{SH} without significantly increasing ρ_{xx} in the SHM layer, which also helps reduce current shunting into the FM layer, which acts as a free layer in SOT-MRAM (see current I_2 in Figure S5). We estimated ρ_{xx} of $Pt_{100.0}Bi_{0.0}$ and $Pt_{100-x}Bi_x$ using four-probe measurements. We prepared two stacks: (a) HR-Si/Ta(2.4 nm)/SiO_2(3 nm) to extract ρ_{xx} of

Ta, and (b) HR-Si/Ta(2.4 nm)/Pt or $Pt_{100-x}Bi_x$(4 nm)/SiO_2(3 nm) to extract ρ_{xx} of Pt and $Pt_{100-x}Bi_x$, by subtracting the contribution of Ta using a parallel resistor model.[3] With 6.0% and 8.7% Bi doping, the power consumption factors reduce to 5.1×10^3 $\mu\Omega$–cm (57.5% reduction) and 7.9×10^3 $\mu\Omega$–cm (34.2% reduction), respectively, compared to 1.2×10^4 $\mu\Omega$–cm for $Pt_{100.0}Bi_{0.0}$ (Figure S5).

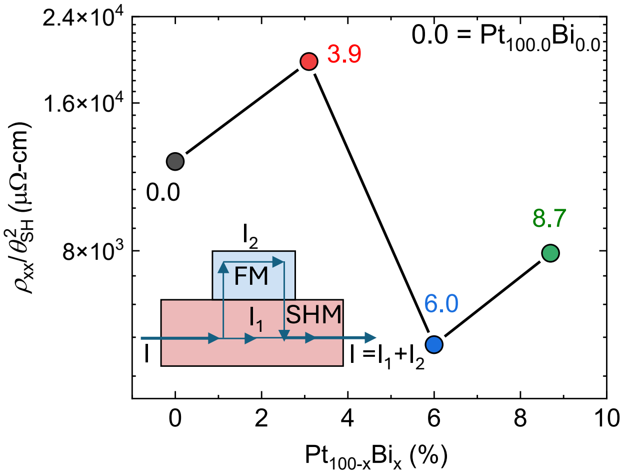

Figure S5. Power consumption ratio (ρ_{xx}/θ_{SH}^2) as a function of Bi impurity in Pt, denoted as $Pt_{100-x}Bi_x$, where x is the weight of Bi. Inset shows the illustration of current shunting into the FM layer in a SOT-MRAM device.

S6: In-plane angular AMR measurements for 100 nm wide SHNO

Figure S6a-c summarizes the in-plane angular anisotropic magnetoresistance (AMR) measurements for all 100 nm wide SHNO, measured at different $\mu_o H_{ext}$ of 100, 150, and 200 mT. The AMR ratio for all the samples is around 0.17-0.26 (see corresponding Figure S6d for 100 nm wide SHNO, respectively). It is typically less than that obtained from microstrips (0.30-0.45), similar to previous reports.[4] However, it increases from 0.15 % ($Pt_{100.0}Bi_{0.0}$) to 0.20 % and 0.18 % for $Pt_{94.0}Bi_{6.0}$ and $Pt_{91.3}Bi_{8.7}$, respectively for 100 nm SHNO, reflecting in changes in SHNO performance, which we will see later in section S9.

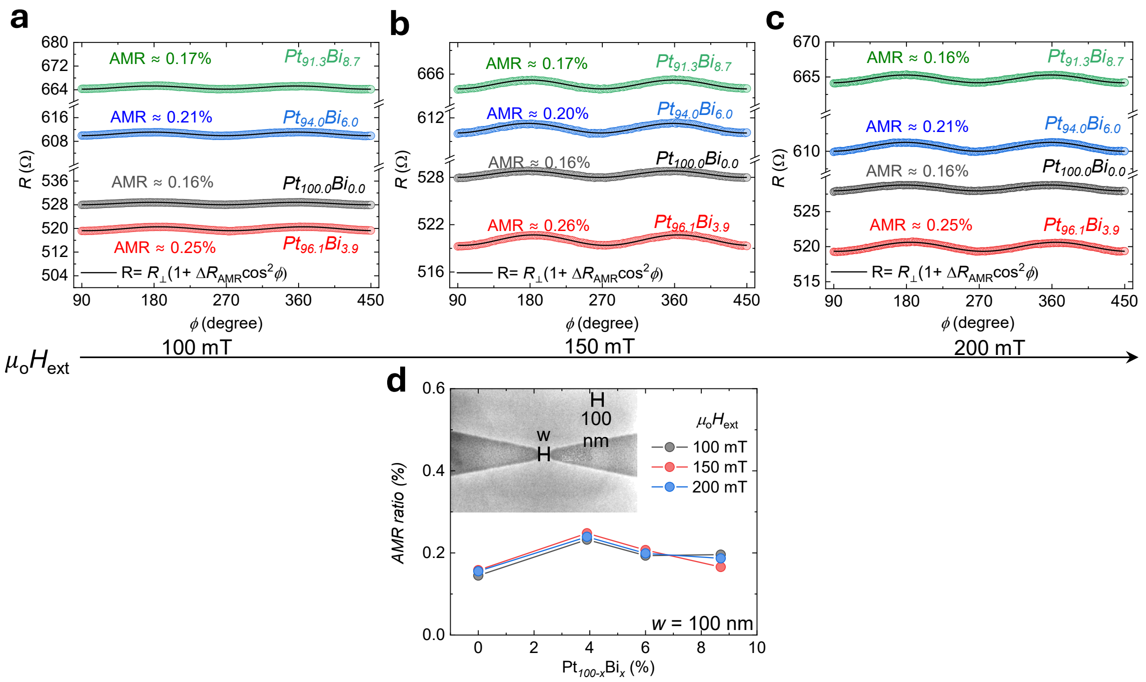

Figure S6. In-plane angular-dependent resistance measurements at 100 mT, 200 mT, and 150 mT for a–c) 100 nm wide SHNOs, fitted using $R = R_{\perp}\left(1 + \Delta R_{\mathrm{AMR}} \cos^2\phi\right)$. AMR ratio as a function of $\mathrm{Pt}_{100-x}\mathrm{Bi}_x$ is shown for d) 100 nm wide SHNO, with SEM image in inset.

S7: Auto-Oscillation at Additional Fields for 100 nm Wide SHNO

Figure S7 shows the PSD generated by the 100 nm wide SHNO at different external magnetic fields of $\mu_0 H_{\mathrm{ext}} = 520$, 560, 600, 640, and 680 mT with $\phi = 20^{\circ}$ and $\theta = 84^{\circ}$ for all $\mathrm{Pt}_{100-x}\mathrm{Bi}_x$ alloys.

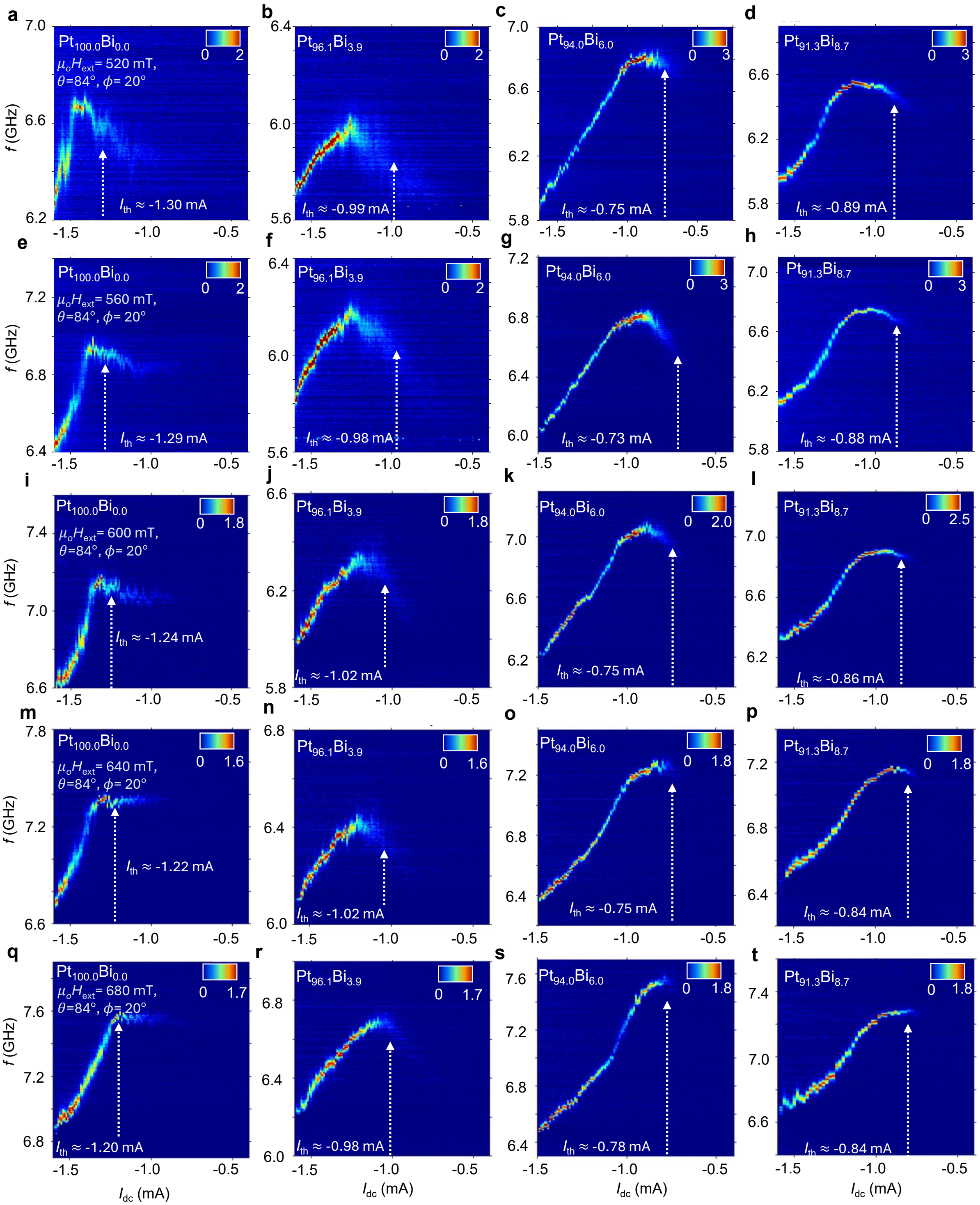

Figure S7. Power spectral density (PSD) generated by the 100 nm wide SHNO as a function of I_{dc}. Panels a–d correspond to $\mu_0 H_{ext} = 520$ mT, e–h to 560 mT, i–l to 600 mT, m–p to 640 mT, and q–t to 680 mT. Panels a, e, i, m, q) $Pt_{100.0}Bi_{0.0}$; b, f, j, n, r) $Pt_{96.1}Bi_{3.9}$; c, g, k, o, s) $Pt_{94.0}Bi_{6.0}$; and d, h, l, p, t) $Pt_{91.3}Bi_{8.7}$. The color bars indicate the peak power in dB relative to the noise floor, while the white arrows serve as a guide to the eye for identifying the threshold current (I_{th}).

S8: Extraction of threshold current

Figure S8a-d illustrates the threshold current estimation for 100 nm wide SHNO. Here, we extract $I_{\rm th}$ via a linear fit of $1/P$ vs. $I_{\rm dc}$, by employing the method by Slavin *et al.*[5] As seen in Figure 4d-g of main text, we find the $I_{\rm th}$ to reduce from -1.30 mA ($Pt_{100.0}Bi_{0.0}$) to -0.95 mA ($Pt_{96.1}Bi_{3.9}$), and then to -0.75 mA ($Pt_{94.0}Bi_{6.0}$) and to -0.89 mA ($Pt_{91.3}Bi_{8.7}$).

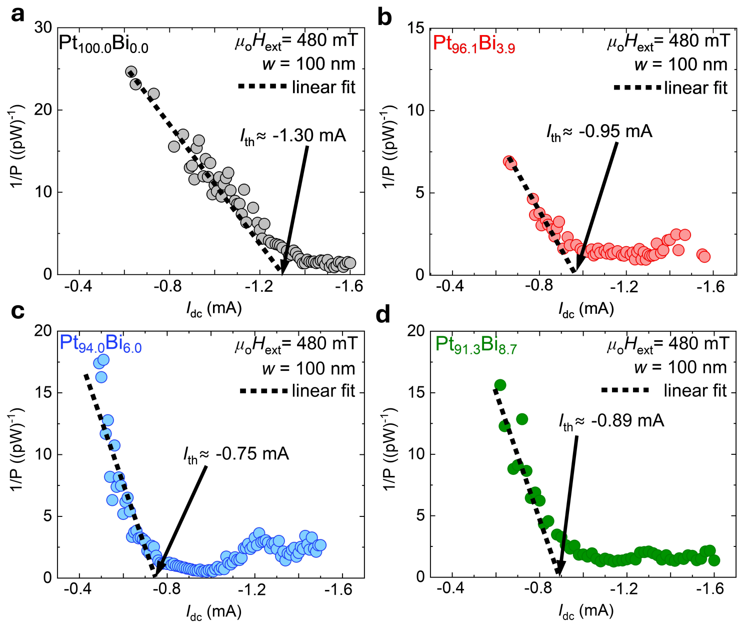

Figure S8. a–d) Inverse power ($1/P$) versus direct current ($I_{\rm dc}$) extracted for 100 nm wide SHNOs. The dashed lines in a–d represent linear fits.

S9: Impact of resistivity, $\rho_{\rm xx}$ of $Pt_{100-x}Bi_x$ alloys on SHNO performance

Figure S9 illustrates the linewidth (Δf) (Figure S9a-d), power (Figure S9e-h), and peak power (Figure S9i-l) for 100 nm wide SHNO extracted from Figure 4d-g from the main text. The higher $\rho_{\rm xx}$ of the PtBi layers, particularly at higher Bi concentrations (see Figure 5a of main text), results in a larger fraction of the applied current being redirected through the $Co_{40}Fe_{40}B_{20}$ layer. This redistribution of current contributes to the enhanced output power (Figure S9e-h), peak output power (Figure S9i-l), and reduced linewidth ($\approx$ 25 MHz) in the $I_{\rm dc}$ range of $-1 \leq I_{\rm dc} \leq -1.6$ mA, (Figure S9a-d) observed in the SHNOs with the

highest Bi content (6.0% and 8.7%), as shown in Figure S9. This effect may be considered a beneficial side effect of Bi incorporation. Interestingly, it also complements the increase in output power due to a a slightly increased AMR ratio at higher Bi content (6.0% and 8.7%) as seen in Section S3 and S6.

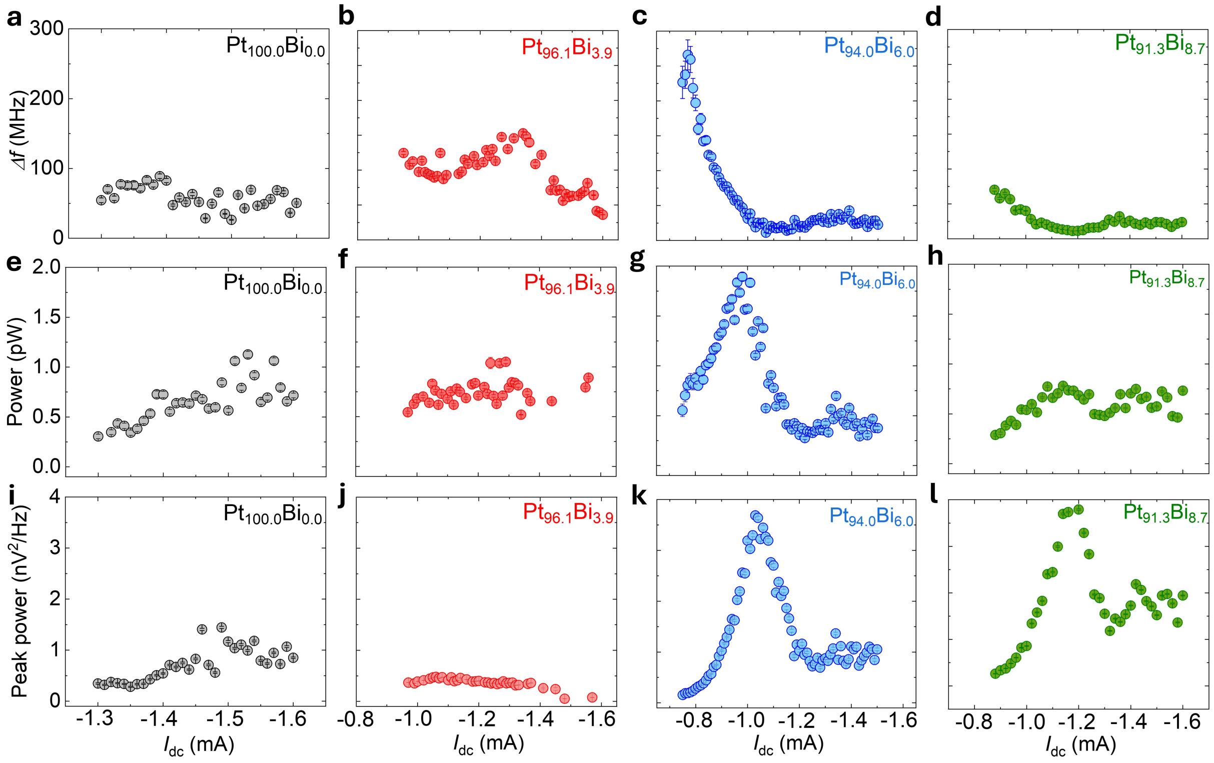

Figure S9. a-d) linewidth (Δf), e-h) power, i-l) peak power for 100 nm wide SHNO extracted from Figure 4d-g of main text for all the $Pt_{100-x}Bi_x$ alloys evaluated in this study along with $Pt_{100.0}Bi_{0.0}$.

S10: Spin mixing conductance, interfacial spin transparency, and their impact on θ_{SH}

The spin Hall efficiency (or spin Hall angle), θ_{SH} (also referred to as ξ_{DL} by some groups), is generally expressed as:[6]

$$\theta_{SH} = T_{int}\,\theta_{SH}^{int}\left[1 - \text{sech}\left(\frac{d}{\lambda_{sd}}\right)\right], \tag{A3}$$

where T_{int} is the interfacial transparency factor and θ_{SH}^{int} is the intrinsic spin Hall angle.

We first determine the effective spin mixing conductance $g_{eff}^{\uparrow\downarrow}$[7,8] from the frequency de-

pendence of the linewidth broadening (Fig. S10a):

$$g_{\text{eff}}^{\uparrow\downarrow} = \left(\frac{\gamma}{2\pi f}\right)\left(\frac{4\pi M_{\text{s}}\, t\, \delta}{g\mu_0\mu_{\text{B}}}\right), \tag{A4}$$

where $\delta = \mu_0 \Delta H_{\text{PtBi or Pt|CoFeB}} - \mu_0 \Delta H_{\text{CoFeB}}$, g is the Landé g-factor, μ_0 is the permeability of free space, t is the thickness of CoFeB, and μ_{B} is the Bohr magneton constant. From $g_{\text{eff}}^{\uparrow\downarrow}$, we extract the real part of the spin mixing conductance:

$$G^{\uparrow\downarrow} = g_{\text{eff}}^{\uparrow\downarrow} \frac{\dfrac{\sigma_{\text{xx}}}{\lambda_{\text{sd}}}\dfrac{h}{2e^2}}{\dfrac{\sigma_{\text{xx}}}{\lambda_{\text{sd}}}\dfrac{h}{2e^2} - g_{\text{eff}}^{\uparrow\downarrow}}, \tag{A5}$$

using $\lambda_{\text{sd}} \propto 1/\rho_{\text{xx}}$ (Elliott–Yafet mechanism).[9]

The resulting $g_{\text{eff}}^{\uparrow\downarrow}$ versus frequency is shown in Fig. S10a, from which the frequency-averaged values (indicated by dashed lines) are used in subsequent analysis. Next, the interfacial spin transparency[10] is evaluated as:

$$T_{\text{int}} = \frac{G^{\uparrow\downarrow}\tanh(d/2\lambda_{\text{sd}})}{G^{\uparrow\downarrow}\coth(d/\lambda_{\text{sd}}) + \dfrac{\sigma_{\text{xx}}}{\lambda_{\text{sd}}}\dfrac{h}{2e^2}}, \tag{A6}$$

which increases with Bi content due to the coupled evolution of σ_{xx} and λ_{sd} (see Fig. S10b).

We therefore report both the lower-bound θ_{SH} from DC-bias ST-FMR and the interface-normalized $\theta_{\text{SH}}/T_{\text{int}}$ as a function of longitudinal resistivity, ρ_{xx} (Fig. S10c). Importantly, $\theta_{\text{SH}}/T_{\text{int}}$ saturates around $x \approx 6 - 8.7\%$ Bi, mirroring the trend of θ_{SH} itself, and the side-jump scaling $\rho_{\text{imp}}^{\text{SH}} \propto \rho_{\text{imp}}^2$ remains preserved after applying T_{int} (compare Fig. 5e and Fig. 5f). Normalizing by T_{int} does not alter the design conclusion that $Pt_{94.0}Bi_{6.0}$ and $Pt_{91.3}Bi_{8.7}$ offers the best compromise between θ_{SH} and ρ_{xx} for low I_{th} and reduced $\rho_{\text{xx}}/\theta_{\text{SH}}^2$, consistent with the AO power, higher Q factor and lower linewidth trends summarized in Figure 6.

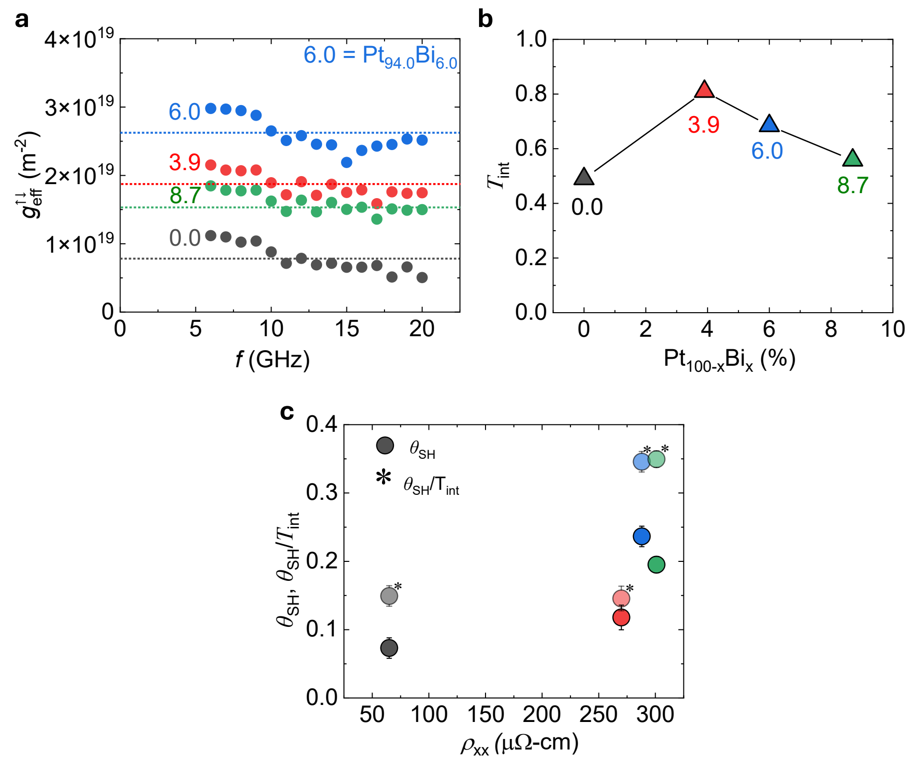

Figure S10. (a) Frequency (f) dependence of $g_{\rm eff}^{\uparrow\downarrow}$ for $\rm Pt_{100.0}Bi_{0.0}$ and $\rm Pt_{100-\mathit{x}}Bi_{\mathit{x}}$ ($x =$ 3.9, 6.0, 8.7). The data points show $g_{\rm eff}^{\uparrow\downarrow}$ extracted from the linewidth broadening at each frequency f, and the dashed lines indicate the frequency-averaged values evaluated from eq. A4. (b) Variation of interfacial spin transparency $T_{\rm int}$ as a function of Bi concentration in $\rm Pt_{100-\mathit{x}}Bi_{\mathit{x}}$ evaluated from eq. A6. (c) Dependence of $\theta_{\rm SH}$ and normalised $\theta_{\rm SH}/T_{\rm int}$ on longitudinal resistivity ρ_{xx}. The asterisks indicate the normalized values $\theta_{\rm SH}/T_{\rm int}$ from eq. A3.

S11: Spin Hall performance of Pt-based alloys

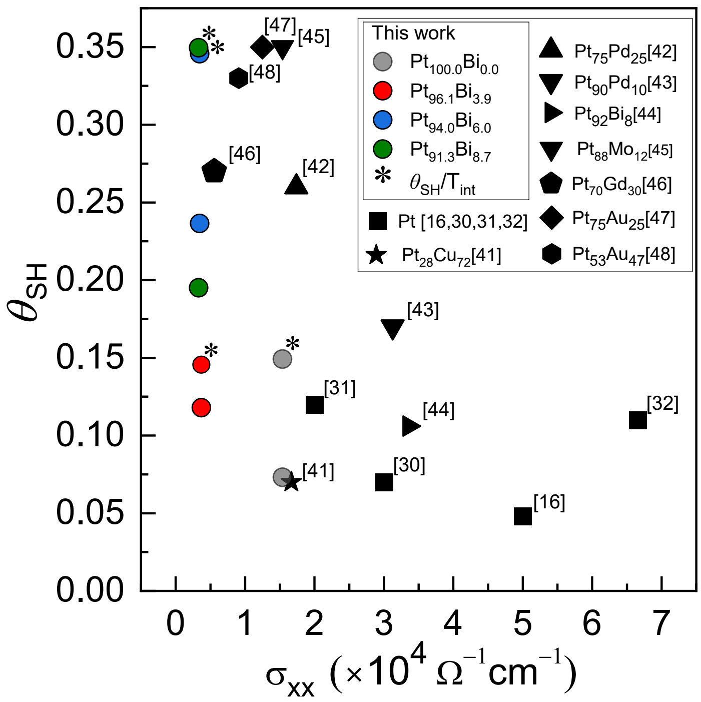

Figure S11. Compilation of θ_{SH} versus σ_{xx} for Pt and Pt-based alloys at room temperature. Symbols denote literature data for Pt–X alloys and $\mathrm{Pt}_{100.0}\mathrm{Bi}_{0.0}$, enabling a direct comparison of alloying trends relevant to spin–orbit torque devices. The numbers in square brackets correspond to references in the main text.

Figure S11 compiles reported room-temperature spin Hall efficiencies θ_{SH} of Pt and Pt-based alloys as a function of longitudinal conductivity σ_{xx}. We highlight representative Pt–X systems studied in the literature, including Pt–Cu, Pt–Pd, Pt–Gd, Pt–Mo, Pt–Au, and Pt–Bi, alongside $\mathrm{Pt}_{100.0}\mathrm{Bi}_{0.0}$, to provide a bird's-eye view of how alloying modifies θ_{SH} across conductivity. This comparison motivates our focus on $\mathrm{Pt}_{100-x}\mathrm{Bi}_x$ compositions.

S12: Mechanism of SHE

The SHE originate from both intrinsic and extrinsic mechanisms, each tied to the material's electronic structure and scattering processes. Intrinsic contributions are dominant in $5d$ transition metals due to strong spin–orbit coupling, which depends on the Berry curvature

of the momentum of conduction electrons in a material, which creates a symmetrical and transverse displacement of the up-spin/down-spin electrons. In contrast, extrinsic mechanisms arise from impurity-induced scattering and are categorized into skew scattering and side-jump scattering.[11] Skew scattering arises from the asymmetric scattering of up-spin and down-spin electrons due to a smaller amount of impurity in the host.[12] Side-jump scattering, on the other hand, arises due to a larger amount of impurity in the host, and occurs when a sudden jump is created near the impurities in the host, leading to a sideways, but symmetrical scattering, similar to intrinsic SHE.[13]

Based on the analogy between AHE and SHE,[14] three different regimes have been widely defined on the basis of the range of σ_{xx}: *(i)* superclean regime ($10^6 < \sigma_{xx} < 10^8\ \Omega^{-1}\mathrm{cm}^{-1}$), *(ii)* good metal regime ($10^4 \lesssim \sigma_{xx} \lesssim 10^6\ \Omega^{-1}\mathrm{cm}^{-1}$), and *(iii)* bad metal/dirty metal regime ($\sigma_{xx} \lesssim 10^4\ \Omega^{-1}\mathrm{cm}^{-1}$). In the superclean regime, SHE is primarily influenced by extrinsic factors, leading to a linear relationship, $\sigma_{\mathrm{SH}}^{\mathrm{xy}} \propto \sigma_{\mathrm{xx}}$. In contrast, in the good metal regime, the intrinsic SHE contribution dominates, making $\sigma_{\mathrm{SH}}^{\mathrm{xy}}$ independent of σ_{xx}. In the bad metal/dirty metal regime, $\sigma_{\mathrm{SH}}^{\mathrm{xy}}$ significantly decreases as σ_{xx} decreases, exhibiting a nonlinear scaling behavior of $\sigma_{\mathrm{SH}}^{\mathrm{xy}} \propto \sigma_{\mathrm{xx}}^{1.6-1.8}$, primarily due to the intrinsic contribution. Additionally, in the good metal regime, there is also an extrinsic SHE component from side-jump scattering, which can cause a deviation from the linear scaling, leading to $\sigma_{\mathrm{SH}}^{\mathrm{xy}} \propto \sigma_{\mathrm{xx}}^2$, influenced by the residual resistivity from impurities. Both the $\sigma_{\mathrm{SH}}^{\mathrm{xy}} \propto \sigma_{\mathrm{xx}}^{1.6-1.8}$ and $\sigma_{\mathrm{SH}}^{\mathrm{xy}} \propto \sigma_{\mathrm{xx}}^2$ can lead to a non-linear scaling of θ_{SH} vs σ_{xx}, (Fig. 5d of main text). Therefore, to confirm the SHE mechanism, we instead focus on the $\rho_{\mathrm{imp}}^{\mathrm{SH}}$ and ρ_{imp}, which are defined as:

$$\rho_{\mathrm{SH}}^{\mathrm{imp}} = \rho_{\mathrm{SH}}^{\mathrm{Pt}_{100-x}\mathrm{Bi}_x} - \rho_{\mathrm{SH}}^{\mathrm{Pt}} \tag{A7}$$

$$\rho_{\mathrm{imp}} = \rho_{\mathrm{Pt}_{100-x}\mathrm{Bi}_x} - \rho_{\mathrm{Pt}} \tag{A8}$$

where $\rho_{\mathrm{SH}}^{\mathrm{Pt}_{100-x}\mathrm{Bi}_x}$ is the spin Hall resistivity of $\mathrm{Pt}_{100-x}\mathrm{Bi}_x$, and $\rho_{\mathrm{SH}}^{\mathrm{Pt}}$ is the spin Hall resistivity of $\mathrm{Pt}_{100.0}\mathrm{Bi}_{0.0}$. Also, $\rho_{\mathrm{Pt}_{100-x}\mathrm{Bi}_x}$ and ρ_{Pt} are the longitudinal resistivity of $\mathrm{Pt}_{100-x}\mathrm{Bi}_x$

and $Pt_{100.0}Bi_{0.0}$, respectively, taken from figure 5a of main text. Since the σ_{xx} does not lie in the superclean regime, we eliminate the possibility of skew scattering in our samples. Therefore, after eliminating the possibilty of skew scattering, we obtain the $\rho_{imp}^{SH} \propto \rho_{imp}^{2}$ trend, confirming the influence of extrinsic side-jump scattering in our samples (see Figure 5e,f in main text).

References

(1) Ou, Y.; Wang, Z.; Chang, C. S.; Nair, H. P.; Paik, H.; Reynolds, N.; Ralph, D. C.; Muller, D. A.; Schlom, D. G.; Buhrman, R. A. Exceptionally high, strongly temperature dependent, spin Hall conductivity of SrRuO3. *Nano Lett.* **2019**, *19*, 3663.

(2) Han, J.; Richardella, A.; Siddiqui, S. A.; Finley, J.; Samarth, N.; Liu, L. Room-temperature spin-orbit torque switching induced by a topological insulator. *Phys. Rev. Lett.* **2017**, *119*, 077702.

(3) Husain, S.; Chen, X.; Gupta, R.; Behera, N.; Kumar, P.; Edvinsson, T.; García-Sánchez, F.; Brucas, R.; Chaudhary, S.; Sanyal, B.; Svedlindh, P.; Kumar, A. Large damping-like spin–orbit torque in a 2D conductive 1T-TaS2 monolayer. *Nano Lett.* **2020**, *20*, 6372–6380.

(4) Behera, N.; Fulara, H.; Bainsla, L.; Kumar, A.; Zahedinejad, M.; Houshang, A.; Åkerman, J. Energy-Efficient $W_{100-x}Ta_x$/Co-Fe-B/MgO Spin Hall Nano-Oscillators. *Phys. Rev. Appl.* **2022**, *18*, 024017.

(5) Tiberkevich, V.; Slavin, A.; Kim, J.-V. Microwave power generated by a spin-torque oscillator in the presence of noise. *Appl. Phys. Lett.* **2007**, *91*.

(6) Liu, L.; Moriyama, T.; Ralph, D. C.; Buhrman, R. A. *Phys. Rev. Lett.* **2011**, *106*, 036601.

(7) Mosendz, O.; Vlaminck, V.; Pearson, J.; Fradin, F.; Bauer, G.; Bader, S.; Hoffmann, A. Detection and quantification of inverse spin Hall effect from spin pumping in permalloy/normal metal bilayers. *Phys. Rev. B* **2010**, *82*, 214403.

(8) Tserkovnyak, Y.; Brataas, A.; Bauer, G. E.; Halperin, B. I. Nonlocal magnetization dynamics in ferromagnetic heterostructures. *Rev. Mod. Phys.* **2005**, *77*, 1375.

(9) Nguyen, M.-H.; Ralph, D.; Buhrman, R. Spin torque study of the spin Hall conductivity and spin diffusion length in platinum thin films with varying resistivity. *Phys. Rev. Lett.* **2016**, *116*, 126601.

(10) Zhang, W.; Han, W.; Jiang, X.; Yang, S.-H.; SP Parkin, S. Role of transparency of platinum–ferromagnet interfaces in determining the intrinsic magnitude of the spin Hall effect. *Nat. Phys.* **2015**, *11*, 496–502.

(11) Niimi, Y.; Otani, Y. Reciprocal spin Hall effects in conductors with strong spin–orbit coupling: a review. *Rep. Prog. Phys.* **2015**, *78*, 124501.

(12) Smit, J. The spontaneous Hall effect in ferromagnetics I. *Physica* **1955**, *21*, 877–887.

(13) Berger, L. Side-jump mechanism for the Hall effect of ferromagnets. *Phys. Rev. B* **1970**, *2*, 4559.

(14) Nagaosa, N.; Sinova, J.; Onoda, S.; MacDonald, A. H.; Ong, N. P. Anomalous Hall effect. *Rev. Mod. Phys.* **2010**, *82*, 1539.
